# Efficacy of oral administration of cystine and theanine in colorectal cancer patients undergoing capecitabine-based adjuvant chemotherapy after surgery: a multi-institutional, randomized, double-blinded, placebo-controlled, phase II trial (JORTC-CAM03)

**DOI:** 10.1007/s00520-019-05205-1

**Published:** 2019-12-06

**Authors:** Reo Hamaguchi, Takashi Tsuchiya, Go Miyata, Toshihiko Sato, Kenichi Takahashi, Koh Miura, Hiroshi Oshio, Hisatsugu Ohori, Keisuke Ariyoshi, Shunsuke Oyamada, Satoru Iwase

**Affiliations:** 1 Department of Palliative Medicine, The Institute of Medical Science, The University of Tokyo, Tokyo, Japan; 2 Present Address: Department of Stress Sciences and Psychosomatic Medicine, Graduate School of Medicine, The University of Tokyo, 7-3-1 Hongo, Bunkyo-ku, Tokyo, 113-8655 Japan; 3 Department of Gastroenterological Surgery, Sendai City Medical Center, Sendai Open Hospital, Sendai, Miyagi, Japan; 4 Department of Gastroenterological Surgery, Iwate Prefectural Central Hospital, Morioka, Iwate, Japan; 5 Department of Surgery, Yamagata Prefectural Central Hospital, Yamagata, Japan; 6 Department of Colorectal Surgery, Tohoku Rosai Hospital, Sendai, Miyagi Japan; 7 Department of Surgery, Miyagi Cancer Center, Sendai, Miyagi Japan; 8 Department of Surgery, Sendai Medical Center, Sendai, Miyagi Japan; 9 Department of Clinical Oncology, Ishinomaki Red Cross Hospital, Ishinomaki, Miyagi Japan; 10 JORTC Data Center, Tokyo, Japan; 11 Present Address: Department of Emergency & Palliative Medicine, Faculty of Medicine, Saitama Medical University, Saitama, Japan

**Keywords:** Cystine and theanine, Capecitabine, Colorectal cancer, Diarrhea, Adverse events, Hand-foot syndrome

## Abstract

**Purpose:**

Capecitabine-based adjuvant chemotherapy for colorectal cancer patients often causes adverse events (AEs), such as diarrhea, stomatitis, anorexia, and hand-foot syndrome (HFS). Cystine and theanine were reported to attenuate some chemotherapy-associated AEs, and hence are also expected to attenuate capecitabine-induced AEs. Therefore, we aimed to investigate the safety and efficacy of cystine/theanine treatment in colorectal cancer patients undergoing capecitabine-based adjuvant chemotherapy after surgery.

**Methods:**

A total of 100 colorectal cancer patients treated with capecitabine as an adjuvant chemotherapy after surgery were randomly allocated into the cystine/theanine group (*n* = 52) or the placebo group (*n* = 48). The primary endpoint was incidence rate of diarrhea of grade 1 or higher in accordance with the Common Terminology Criteria for AEs (CTCAE) v.4.0, Japanese Clinical Oncology Group (JCOG) version. The secondary endpoints included incidence rates of other AEs (CTCAE v.4.0-JCOG), as well as the incidence rate of HFS according to the HFS grading scale.

**Results:**

There were no significant differences in capecitabine-induced AEs between the two groups. However, the incidence rate of diarrhea of grade 1 or higher tended to be lower in the cystine/theanine group than the placebo group (18.4% vs. 28.9%, *p* = 0.169) as well as the incidence rate of HFS of grade 1 or higher (CTCAE v.4.0-JCOG or HFS grading scale) (67.4% vs. 77.8%, *p* = 0.185, 67.3% vs. 80.0%, *p* = 0.124, respectively).

**Conclusion:**

This trial demonstrated that cystine/theanine treatment of colorectal cancer patients undergoing capecitabine-based adjuvant chemotherapy after surgery is safe and has the tendency to reduce the incidence rate of diarrhea or HFS.

**Trial registration:**

UMIN000024784

**Electronic supplementary material:**

The online version of this article (10.1007/s00520-019-05205-1) contains supplementary material, which is available to authorized users.

## Background

For locally advanced colorectal cancers, combined chemotherapy after surgery is usually recommended. In Japan, capecitabine therapy is a standard adjuvant oral chemotherapy for clinical stage III colorectal cancer [1–4]. However, adverse events (AEs), such as hand-foot syndrome (HFS) [5], as well as gastrointestinal symptoms (stomatitis, anorexia, diarrhea, etc.), bone marrow suppression, and an increase in hepatobiliary enzyme levels [6] are often caused by capecitabine therapy. These AEs negatively affect both treatment course and a patient’s quality of life (QOL). Withdrawal or a reduced dosage of capecitabine with symptomatic treatment is often required, because there are no standard prevention therapies for these AEs. Therefore, a reduction or prevention therapy for these AEs, which will improve the completion rate of capecitabine therapy, is urgently required.

Cystine/theanine is a supplement that contains l-cystine (700 mg) and l-theanine (280 mg), which are known to be precursors associated with the synthesis of glutathione (GSH). Cystine is a dipeptide of the sulfur amino acid cysteine and is reduced and converted to cysteine in the cell. Theanine is also an amino acid and is metabolized to glutamic acid and ethylamine. Cysteine and glutamic acid are synthesized to GSH in humans. GSH is a substrate in conjugation reactions for detoxification, and works as a vital substance for antioxidation reactions that reduce reactive oxygen species, such as peroxides and free radicals. Glutamine, which is also a precursor of GSH and is similar to cystine/theanine, is reported to have a positive effect in preventing and treating oral mucositis in patients receiving mucotoxic cancer chemotherapy [7]. In addition, GSH was reported to show neuroprotective effects for patients undergoing oxaliplatin-based chemotherapy [8]. It was also reported that perioperative oral administration of cystine/theanine suppressed inflammation and enhanced recovery after surgery [9]. Moreover, previous research demonstrated that the incidence rate of diarrhea induced by S-1 adjuvant chemotherapy in colorectal cancer patients after surgery was significantly less in the cystine/theanine group (4.5%) than in the control group (41.7%) [10]. It was also demonstrated that the completion rate of the first course of S-1 at the prescribed dose was significantly higher in the cystine/theanine group (90.9%) than in the control group (41.7%). These results of previous studies support the hypothesis that cystine/theanine will attenuate AEs and improve the QOL of patients treated with capecitabine therapy.

Therefore, we conducted a randomized, double-blinded, placebo-controlled study to investigate the efficacy of cystine/theanine therapy in colorectal cancer patients undergoing capecitabine therapy as an adjuvant chemotherapy after surgery.

## Methods

### Patients and study design

The design and rationale of this trial have been published previously [11]. This trial was a multi-institutional, randomized, double-blinded, placebo-controlled, phase II trial investigating the safety and efficacy of oral administration of cystine/theanine in colorectal cancer patients undergoing capecitabine-based adjuvant chemotherapy after surgery. This trial was performed in 7 participating institutions located in Japan, as follows: Sendai Open Hospital, Iwate Prefectural Central Hospital, Yamagata Prefectural Central Hospital, Tohoku Rosai Hospital, Miyagi Cancer Center, Sendai Medical Center, and Ishinomaki Red Cross Hospital.

Eligible patients were enrolled in the Japanese Organisation for Research and Treatment of Cancer (JORTC) data center using a web-based electronic data-capturing system. Patients were randomly assigned to receive either cystine/theanine or placebo, using stratified permuted block randomization methods. Random assignment was performed by a computer-generated randomization schedule with a 1:1 allocation ratio, balanced for the following stratification factors: (1) study site, (2) age (< 71 or ≥ 71 years), and (3) site of the cancer (colon or rectum). Patients and investigators responsible for treatment were blinded to the administered reagent (cystine/theanine or placebo). A unique identification (ID) number was allocated to each patient at enrollment. Data were analyzed by ID number only. The master list linking personal information of the participants to ID numbers was maintained in a separate locked cabinet and a password-protected hard drive at each institution.

All patients provided written informed consent before trial enrollment. All study procedures were in accordance with the Declaration of Helsinki and the Japanese ethical guidelines for clinical research. The JORTC Protocol Review Committee and the Institutional Review Board of all participating institutions approved this study protocol. This trial has been registered at the University hospital Medical Information Network (UMIN) Clinical Trials Registry as UMIN000024784 on November 10, 2016. Recruitment of study patients was initiated on November 22, 2016, and was completed on July 10, 2018. This study followed the Standard Protocol Items for Randomized Trials 2013 statement and its checklist [12].

### Eligibility

Eligible patients were 20 to 80 years of age, had histologically confirmed colorectal cancer, agreed to undergo adjuvant capecitabine therapy after surgery, had Eastern Cooperative Oncology Group (ECOG) Performance Status ≤ 1, and had laboratory findings within the following ranges (at ≤ 14 days prior to enrollment): leukocytes ≥ 3000/mm^3^, platelets ≥ 75,000/mm^3^, hemoglobin ≥ 9.0 g/dL, total bilirubin ≤ 2.0 mg/dL, aspartate aminotransferase ≤ 100 U/L, alanine aminotransferase ≤ 100 U/L, and serum creatinine ≤ 1.5 mg/dL.

We excluded patients with a history of chemotherapy or radiation therapy, those who were taking dietary supplements containing amino acids or protein (except if they had discontinued more than 7 days before the start of the study), those who had a history of previous administration of cystine or theanine, those who were taking dietary supplements derived from herbs (such as St. John’s wort or others) except for multivitamins, those who were pregnant or breastfeeding, those who had immune deficiency or phenylketonuria, those who were enrolled in other clinical trials, those who had a cognitive disorder prior to enrollment, those who had a psychiatric disorder that may affect data sampling, those who were unable to communicate verbally, those who were uncapable of oral ingestion, those who had malabsorption syndrome, those who had a history of gastrectomy, those who had active gastrointestinal bleeding unrelated with the cancer, and those who the investigator considered were not suitable for the study.

### Assessment

Baseline patient characteristics, such as age, sex, histology of colorectal cancer, cancer stage, ECOG scale of performance status, height, weight, and current treatment, were collected. AEs were graded according to the Common Terminology Criteria for Adverse Events (CTCAE) v.4.0, Japanese Clinical Oncology Group (JCOG) version. HFS was also assessed by the HFS grading scale suggested by Blum JL, et al. (Supplemental Table 1) [11, 13]. Health-associated quality of life was assessed according to the Japanese version of the European Organisation for Research and Treatment of Cancer (EORTC) Quality of Life Questionnaire module for all cancer patients (QLQ-C30) and for colorectal cancer patients (QLQ-CR29).

### Treatment and interventions

Adjuvant capecitabine therapy after surgery was conducted in accordance with the prescribing instructions [6], as follows: 1250 mg/m^2^ twice daily orally (morning and evening; equivalent to 2500 mg/m^2^ total daily dose) for 2 weeks followed by a 1-week rest period in 3-week cycles for a total of 8 cycles (24 weeks). The time between surgery and the start of capecitabine treatment was not precisely set and was left to the decision of each participating institution.

Cystine/theanine, which contains l-cystine (700 mg), l-theanine (280 mg), and other components (maltitol, aspartame, and citric acid), or placebo, which contains palatinose (833 mg), powdery starch syrup (147 mg), and other components (maltitol, aspartame, and citric acid), was administered in 1 dose every morning from the start of capecitabine therapy until completion of 4 courses of capecitabine therapy (Supplemental Fig 1).

Supportive treatments for HFS were allowed in this study. There is no standard prevention for HFS; however, moisturization is considered to be important as a prophylactic treatment for HFS in clinical settings [14]. Moisturizing therapy with urea ointment (Urepearl®, Keratinamin®, Pastaron®, etc.) as a supportive treatment for HFS was applied from the start of capecitabine therapy. Other drugs or treatments for HFS were also allowed, and their durations and reasons for use were recorded.

### Endpoints

The primary endpoint was incidence rate of diarrhea of grade 1 or higher. Secondary endpoints also included incidence rate of other AEs, such as HFS of grade 1 or higher (HFS grading scale); diarrhea of grade 2 or higher; other AEs of grade 1 or higher, grade 2 or higher; EORTC QLQ-C30 and EORTC QLQ-CR29 scores; adherence to protocol; completion rate of 4 courses of capecitabine therapy, and the proportion of completion without delay or dose reduction; time to completion (4 courses of capecitabine therapy); and total dose of capecitabine.

### Statistical analysis

Sample size calculations were made based on a previous study [10]. The incidence rate of diarrhea induced by adjuvant S-1 therapy in colorectal cancer patients after surgery was significantly less in the cystine/theanine group (4.5%) than in the control group (41.7%). Capecitabine is an oral pyrimidine fluoride anticancer drug similar to S-1, and the incidence rate of diarrhea after capecitabine monotherapy according to the prescribing information is 36.8% [6]. Therefore, it was estimated that the incidence rate of diarrhea after capecitabine therapy would be 5% with cystine/theanine and 25% with placebo [11]. Assuming a 10% dropout rate, we calculated 50 patients for each group (100 samples in total) with a one-sided significance level of 5% and a power of 80%.

All statistical procedures were detailed in the statistical analysis plan before data evaluation. The efficacy and safety analysis population comprised all randomized patients who had received at least 1 administration of protocol treatment. Patients who were found to be ineligible after randomization were excluded from the analysis of efficacy, although they were included in the analysis of safety. The incidence rates of AEs were analyzed using a one-sided Fisher exact test at a significance level of 5% according to the intention-to-treat principle. Point estimates and 90% confidence intervals for differences between 2 groups were calculated. Means and standard deviations (SD) were calculated for adherence (%) to cystine/theanine therapy or placebo. EORTC QLQ-C30 and QLQ-CR29 scores were calculated at each course of capecitabine treatment and analyzed using a two-sided *t* test at a significance level of 10%. In this analysis, a complete case analysis and last observation carried forward analysis were performed. The completion rate of 4 courses of capecitabine therapy and the proportion of completion without delay or dose reduction were analyzed with a two-sided chi-square test at a significance level of 10%. Time to completion (4 courses of capecitabine therapy) and the total dose of capecitabine were analyzed using a two-sided *t* test at a significance level of 10%. Data were analyzed using SAS version 9.4 software (SAS Institute Inc., Cary, NC, USA).

## Results

### Patient populations

**Fig. 1:**
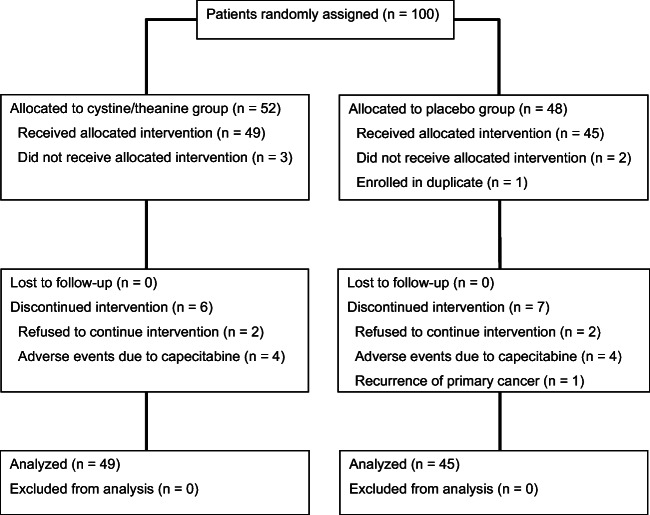
CONSORT diagram. Flow diagram of the number of participants who were randomly assigned to the cystine/theanine group or the placebo group, and were analyzed, is shown

A total of 100 patients from 7 participating institutions were randomly assigned to either the cystine/theanine group (*n* = 52) or the placebo group (*n* = 48) (Fig. 1). In the cystine/theanine group, 3 patients who did not receive the planned treatment were excluded. In the placebo group, 2 patients who did not receive the planned treatment and 1 patient enrolled in duplicate by mistake were excluded. Therefore, efficacy analysis and safety analysis were conducted on 49 patients in the cystine/theanine group and 45 patients in the placebo group. Patient characteristics are shown in Table 1. The 2 groups were well balanced at baseline.

**Table 1:** Baseline characteristics of the patients

Characteristics	No. of patients (%)	
	Cystine/theanine (*n* = 52)	Placebo (*n* = 48)
Age, years		
Median (range)	63.5 (36–79)	65.5 (46–80)
20 ≤ age < 71	38 (73.1)	35 (72.9)
71 ≤ age ≤ 80	14 (26.9)	13 (27.1)
Sex		
Male	31 (59.6)	25 (52.1)
Female	21 (40.4)	23 (47.9)
Height, median (range)	163 (144.2–179.0)	160.1 (144.0–181.1)
Body weight, median (range)	60.1 (37.0–123.5)	56.4 (37.2–95.3)
ECOG performance status		
0	52 (100)	47 (97.9)
1	0 (0.0)	1 (2.1)
Tumor location		
Colon	39 (75.0)	35 (72.9)
Rectum	13 (25.0)	13 (27.1)
Histology*		
Papillary adenocarcinoma	1 (1.9)	0 (0.0)
Tubular adenocarcinoma		
Well-differentiated type	10 (19.2)	6 (12.5)
Moderately differentiated type	34 (65.4)	37 (77.1)
Poorly differentiated adenocarcinoma		
Solid type	1 (1.9)	0 (0.0)
Nonsolid type	2 (3.8)	0 (0.0)
Mucinous adenocarcinoma	0 (0.0)	0 (0.0)
Signet ring cell carcinoma	0 (0.0)	0 (0.0)
Medullary carcinoma	0 (0.0)	0 (0.0)
Adenosquamous carcinoma	0 (0.0)	0 (0.0)
Squamous cell carcinoma	0 (0.0)	0 (0.0)
Other carcinoma	0 (0.0)	3 (6.3)
Clinical stage		
II	17 (32.7)	11 (22.9)
IIIa	30 (57.7)	30 (62.5)
IIIb	2 (3.8)	5 (10.4)
Institutions		
Sendai Open Hospital	26 (50)	23 (47.9)
Iwate Prefectural Central Hospital	6 (11.5)	7 (14.6)
Yamagata Prefectural Central Hospital	8 (15.4)	8 (16.7)
Tohoku Rosai Hospital	0 (0.0)	2 (4.2)
Miyagi Cancer Center	1 (1.9)	0 (0.0)
Sendai Medical Center	9 (17.3)	8 (16.7)
Ishinomaki Red Cross Hospital	2 (3.8)	0 (0.0)

*ECOG* Eastern Cooperative Oncology Group

*Japanese Classification of Colorectal, Appendiceal, and Anal Carcinoma, Ninth Edition (Japanese Society for Cancer of the Colon and Rectum)

### Efficacy and safety assessment

Adherence to cystine/theanine treatment or placebo was good and did not differ remarkably between the 2 groups. The total adherence to cystine/theanine therapy or placebo was 99.4% and 97.5%, respectively (Supplemental Table 2).

The incidence rates of diarrhea of grade 1 or higher and grade 2 or higher are shown in Table 2. Although there were no significant differences between the 2 groups, the incidence rate of diarrhea of grade 1 or higher had a tendency to be lower in the cystine/theanine group than in the placebo group (18.4% vs. 28.9%, *p* = 0.169).

**Table 2:** Incidence rates of diarrhea of grade 1 or higher and grade 2 or higher (CTCAE v.4.0-JCOG)

		No. of patients (%)		Difference between groups
		Cystine/theanine (*n* = 49)	Placebo (*n* = 45)	Point estimation [90% CI], *p* value
Diarrhea	Grade ≥ 1	9 (18.4)	13 (28.9)	− 10.50 [− 27.29, 6.66], 0.169
	Grade ≥ 2	5 (10.2)	7 (15.6)	− 5.40 [− 22.19, 11.74], 0.320

*CTCAE v.4.0-JCOG* Common Terminology Criteria for Adverse Events version 4.0-Japanese Clinical Oncology Group version

The incidence rate of AEs of either grade 1 or higher or grade 2 or higher is shown in Table 3. The incidence rate of HFS (HFS grading scale) is shown in Table 4. There were no significant differences between the 2 groups. However, the incidence rate of HFS of grade 1 or higher had a tendency to be lower in the cystine/theanine group than the placebo group (67.4% vs. 77.8%, *p* = 0.185 [using CTCAE v.4.0-JCOG], and 67.3% vs. 80.0%, *p* = 0.124 [using the HFS grading scale]). The proportion of patients receiving supportive treatment for HFS, as summarized in Table 5, was 83.7% in the cystine/theanine group and 86.7% in the placebo group.

**Table 3:** Incidence rates of adverse events without diarrhea of grade 1 or higher and grade 2 or higher (CTCAE v.4.0-JCOG)

		No. of patients (%)		Difference between groups
		Cystine/theanine (*n* = 49)	Placebo (*n* = 45)	Point estimation [90% CI], *p* value
Hand-foot syndrome*	Grade ≥ 1	33 (67.4)	35 (77.8)	− 10.40 [− 27.05, 6.88], 0.185
	Grade ≥ 2	20 (40.8)	20 (44.4)	− 3.60 [− 20.46, 13.64], 0.442
Nausea	Grade ≥ 1	8 (16.3)	6 (13.3)	3.00 [− 13.98, 19.95], 0.756
	Grade ≥ 2	2 (4.1)	2 (4.4)	− 0.40 [− 17.48, 16.57], 0.659
Vomiting	Grade ≥ 1	2 (4.1)	2 (4.4)	− 0.40 [− 17.48, 16.57], 0.659
	Grade ≥ 2	1 (2.0)	1 (2.2)	− 0.20 [− 17.28, 16.83], 0.731
Anorexia	Grade ≥ 1	11 (22.5)	9 (20.0)	2.40 [− 14.61, 19.46], 0.705
	Grade ≥ 2	1 (2.0)	4 (8.9)	− 6.80 [− 23.78, 10.12], 0.155
Mucositis oral	Grade ≥ 1	16 (32.7)	14 (31.1)	1.50 [− 15.65, 18.28], 0.648
	Grade ≥ 2	3 (6.1)	3 (6.7)	− 0.50 [− 17.65, 16.29], 0.620
Constipation	Grade ≥ 1	3 (6.1)	3 (6.7)	− 0.50 [− 17.65, 16.29], 0.620
	Grade ≥ 2	No data	No data	
Malaise	Grade ≥ 1	12 (24.5)	9 (20.0)	4.50 [− 12.64, 21.43], 0.779
	Grade ≥ 2	3 (6.1)	2 (4.4)	1.70 [− 15.48, 18.52], 0.792
Abdominal pain	Grade ≥ 1	7 (14.3)	4 (8.9)	5.40 [− 11.74, 22.19], 0.872
	Grade ≥ 2	1 (2.0)	No data	2.00 [− 15.09, 19.03], 1.000
Skin and subcutaneous tissue disorders	Grade ≥ 1	7 (14.3)	5 (11.1)	3.20 [− 13.89, 20.08], 0.778
	Grade ≥ 2	1 (2.0)	2 (4.4)	− 2.40 [− 19.46, 14.61], 0.468
Skin hyperpigmentation	Grade ≥ 1	27 (55.1)	20 (44.4)	10.70 [− 6.66, 27.51], 0.892
	Grade ≥ 2	2 (4.1)	No data	4.10 [− 13.10, 20.99], 1.000
Fever	Grade ≥ 1	1 (2.0)	1 (2.2)	− 0.20 [− 17.28, 16.83], 0.731
	Grade ≥ 2	No data	No data	
Decreased white blood cell count	Grade ≥ 1	13 (26.5)	11 (24.4)	2.10 [− 15.09, 19.03], 0.680
	Grade ≥ 2	12 (24.5)	8 (17.8)	6.70 [− 10.40, 23.60], 0.852
Decreased neutrophil count	Grade ≥ 1	27 (55.1)	21 (46.7)	8.40 [− 8.89, 25.35], 0.847
	Grade ≥ 2	14 (28.6)	12 (26.7)	1.90 [− 15.30, 18.79], 0.668
Anemia	Grade ≥ 1	17 (34.7)	17 (37.8)	− 3.10 [− 20.08, 13.98], 0.461
	Grade ≥ 2	1 (2.0)	3 (6.7)	− 4.60 [− 21.63, 12.37], 0.277
Decreased platelet count	Grade ≥ 1	13 (26.5)	14 (31.1)	− 4.60 [− 21.63, 12.37], 0.396
	Grade ≥ 2	No data	No data	
Increased AST	Grade ≥ 1	18 (36.7)	6 (13.3)	23.40 [6.21, 39.45], 0.998
	Grade ≥ 2	No data	No data	
Increased ALT	Grade ≥ 1	15 (30.6)	5 (11.1)	19.50 [2.41, 35.83], 0.996
	Grade ≥ 2	No data	No data	
Increased blood bilirubin	Grade ≥ 1	13 (26.5)	7 (15.6)	11.00 [− 6.16, 27.70], 0.941
	Grade ≥ 2	3 (6.1)	1 (2.2)	3.90 [− 13.30, 20.73], 0.931
Increased ALP	Grade ≥ 1	9 (18.4)	5 (11.1)	7.30 [− 9.79, 24.08], 0.900
	Grade ≥ 2	No data	No data	
Increased GGT	Grade ≥ 1	15 (30.6)	7 (15.6)	15.10 [− 2.16, 31.59], 0.976
	Grade ≥ 2	1 (2.0)	No data	2.00 [− 15.09, 19.03], 1.000
Increased creatinine	Grade ≥ 1	28 (57.1)	22 (48.9)	8.30 [− 9.09, 25.12], 0.843
	Grade ≥ 2	2 (4.1)	1 (2.2)	1.90 [− 15.30, 18.79], 0.863

*CTCAE v.4.0-JCOG* Common Terminology Criteria for Adverse Events version 4.0-Japanese Clinical Oncology Group version, *AST* aspartate aminotransferase, *ALT* alanine aminotransferase, *ALP* alkaline phosphatase, *GGT* γ-glutamyltransferase

*Described as palmar-plantar erythrodysesthesia syndrome according to CTCAE v.4.0-JCOG

**Table 4:** Incidence rates of hand-foot syndrome

		No. of patients (%)		Difference between groups
		Cystine/theanine (*n* = 49)	Placebo (*n* = 45)	Point estimation [90% CI], *p* value
Hand-foot syndrome	Grade ≥ 1	33 (67.3)	36 (80.0)	− 12.7 [− 29.22, 4.65], 0.124
	Grade ≥ 2	19 (38.8)	19 (42.2)	− 3.5 [− 20.26, 13.77], 0.448
	Grade 3	5 (10.2)	5 (11.1)	− 0.9 [− 17.94, 16.03], 0.574

Hand-foot syndrome grading scale modified from [13]

**Table 5:** Supportive treatments for hand-foot syndrome

	No. of patients (%)	
	Cystine/theanine (*n* = 49)	Placebo (*n* = 45)
Supportive treatments for HFS	41 (83.7)	39 (86.7)
Topical medicine		
Urea ointment	34 (69.4)	29 (64.4)
Other than urea ointment*	21 (42.9)	20 (44.4)
Oral medicine**	3 (6.1)	4 (8.9)
Others***	1 (2.0)	3 (6.7)

*Steroid ointment and/or moisturizing ointment

**Vitamin B_6_

***Moisturizing ointment from drugstore

EORTC QLQ-C30 and EORTC QLQ-CR29 scores were calculated. Although complete case analysis and last observation carried forward analysis showed that the cystine/theanine group had tendencies of less constipation symptoms and better cognitive function than the placebo group, there were no significant differences between the 2 groups (Supplemental Table 3 and Table 4).

The completion rate of 4 courses of capecitabine treatment without delay or dose reduction, time to completion (4 courses of capecitabine therapy), and total dose of capecitabine were investigated and there were no significant differences between the 2 groups (Supplemental Table 5, Table 6, and Table 7).

## Discussion

To the best of our knowledge, this is the first multi-institutional, randomized, double-blinded, placebo-controlled, phase II trial to investigate the efficacy of cystine/theanine in colorectal cancer patients undergoing capecitabine-based adjuvant chemotherapy after surgery. This study demonstrated that cystine/theanine was safe for colorectal cancer patients receiving adjuvant capecitabine therapy after surgery, and had the tendency to reduce the incidence rate of diarrhea and HFS. This study also showed good adherence to cystine/theanine treatment.

Diarrhea is a common AE induced by capecitabine treatment. Although probiotics or loperamide are used as symptomatic treatments for diarrhea, there are no standard prophylactic treatments, and withdrawal or reduction of the prescribe dosage of capecitabine treatment is often required [6]. A randomized phase III trial investigating the efficacy of long-acting release octreotide in patients with colorectal cancer for the prevention of diarrhea induced by chemotherapy (comprising fluorouracil, capecitabine, and/or irinotecan) failed to confirm any positive effects [15]. The present study did not demonstrate that the cystine/theanine group was significantly superior to the placebo group regarding capecitabine-associated AEs; however, the incidence rate of diarrhea of grade 1 or higher had a tendency to be lower in the cystine/theanine group than in the placebo group (Table 2). Considering that cystine/theanine is a supplementary treatment, we believe that a reduction of 10% or more in diarrhea compared to the control group may have a clinically meaningful effect.

HFS is the most common AE induced by capecitabine treatment, and it remains unclear as to how HFS develops. Although it was reported that oral pyridoxine or the cox-2 inhibitor celecoxib showed the possibility of an ameliorating effect on HFS, a standard oral preventive therapy for HFS has not yet been established [16–19]. Nonpharmacological interventions, such as moisturization, avoidance of pressure, prevention of injury, and elevation or cooling of the hands and feet, are recommended for the relief of symptoms [14]. Therefore, topical moisturizing agents or steroids are commonly used as pharmacological interventions as a prophylactic treatment for HFS. A urea cream that is used for the treatment of hyperkeratotic conditions, such as dry skin, rough skin, and dermatitis, was reported to be superior for capecitabine-associated HFS to a medical ointment containing antioxidants and nourishing oil extracts (Mapisal®; Medac GmbH, Wedel, Germany) in a randomized comparative study [20]. The present study demonstrated that the incidence rate of HFS of grade 1 or higher (CTCAE v.4.0-JCOG or HFS grading scale) had a tendency to be lower in the cystine/theanine group than in the placebo group (Table 3, Table 4). In this study, the use of supportive treatments, including urea ointment for hand-foot syndrome, did not differ between the cystine/theanine group and the placebo group. Although further studies are required, we speculate that a reduction of 10% or more in HFS in the cystine/theanine group compared with the control group indicates a clinically positive effect, as there are no standard promising prophylactic treatments for HFS.

Cystine and theanine are amino acids that are contained in various foods, and they are widely used as food additives. Moreover, previous studies of humans reported no severe AEs caused by cystine/theanine [21, 22]. This study showed that there were no significant increases in the incidence rate of AEs of grade 1 or higher, as well as grade 2 or higher, associated with the cystine/theanine group compared with the placebo group. The results of this study were consistent with previous reports and showed the safety of cystine/theanine in colorectal cancer patients undergoing capecitabine-based adjuvant chemotherapy after surgery [21, 22].

We acknowledge that this study has several limitations. First, the sample size calculation was based on a previous study on the effects of cystine/theanine on S-1-induced diarrhea [10], because we had no data of the effects of cystine/theanine on capecitabine-induced AEs. Therefore, although capecitabine is an oral pyrimidine fluoride anticancer drug similar to S-1, and the incidence rate of diarrhea after capecitabine monotherapy is also close to that after S-1 monotherapy, our results should be interpreted with caution. Moreover, differences in the study design between this previous study and the present study may have contributed to our results. In the previous study, cystine/theanine was administered 1 week before the start of S-1 treatment as an adjuvant chemotherapy after surgery, whereas in our study, cystine/theanine was administered at the start of capecitabine treatment. Therefore, more samples may be required to investigate the efficacy of cystine/theanine on capecitabine-induced AEs. Second, patients with a poorer performance status were not enrolled, and hence, the effects of cysteine/theanine on the AEs of these patients require additional investigation.

## Conclusion

We performed the first multi-institutional, randomized, double-blinded, placebo-controlled, phase II trial to investigate the efficacy of cystine/theanine in colorectal cancer patients undergoing capecitabine-based adjuvant chemotherapy after surgery, and demonstrated that cystine/theanine was safe and has the tendency to reduce the incidence rate of diarrhea and HFS. A phase III trial is required to confirm the effects of cystine/theanine on capecitabine-induced diarrhea or HFS.

## Electronic supplementary material

ESM 1 (PPTX 61 kb).

ESM 2 (DOCX 202 kb).
