# Supplementary material for: Efficacy of oral administration of cystine and theanine in colorectal cancer patients undergoing capecitabine-based adjuvant chemotherapy after surgery: a multi-institutional, randomized, double-blinded, placebo-controlled, phase II trial (JORTC-CAM03)
Source: Support Care Cancer. 2019 Dec 6;28(8):3649–57. doi: 10.1007/s00520-019-05205-1 (PMC7316838; doi:10.1007/s00520-019-05205-1)
Supplement: Supplementary file 1 — (PPTX 61 kb). [file 520_2019_5205_MOESM1_ESM.pptx]

## Slide 1
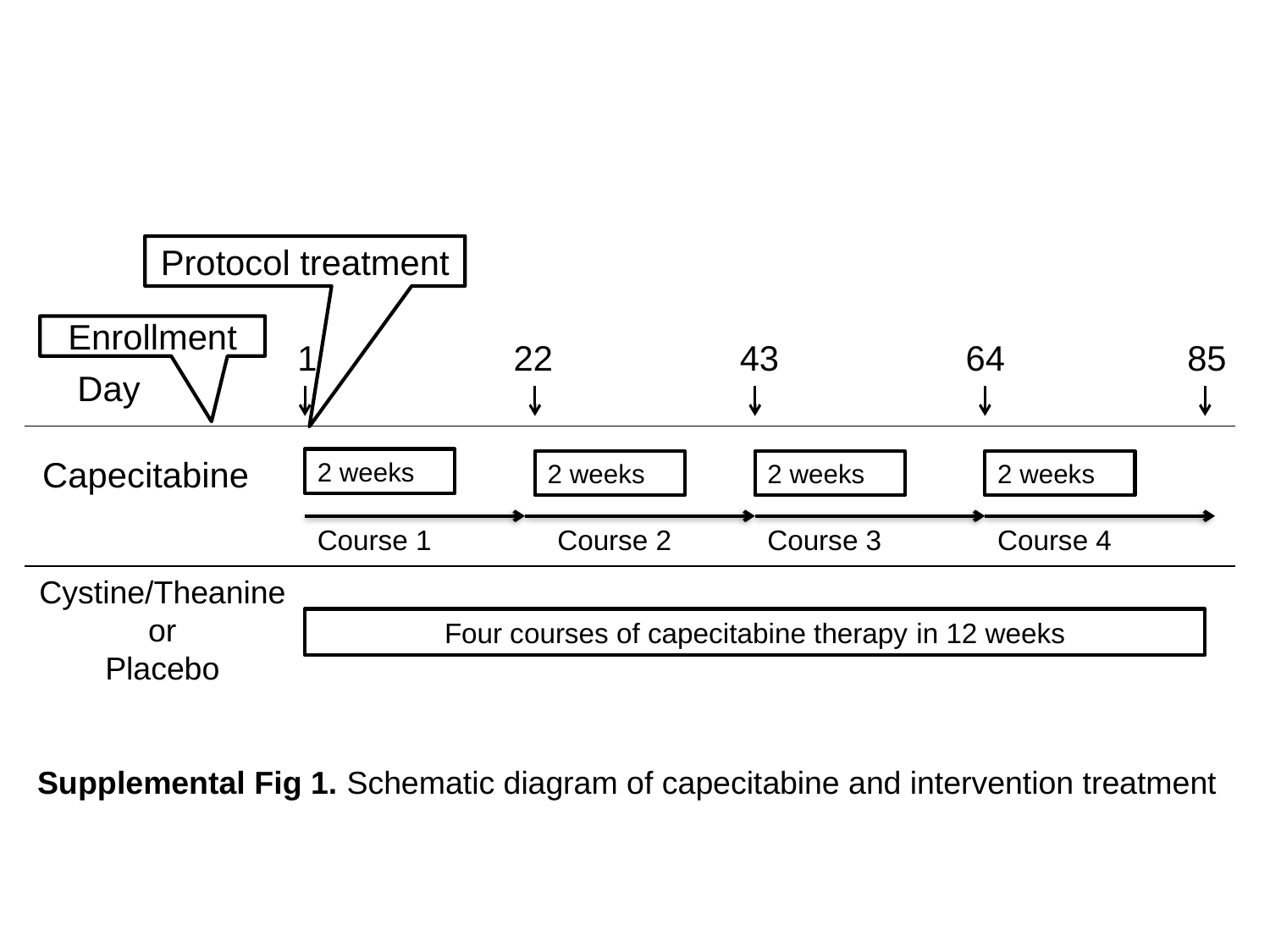

Protocol treatment
Enrollment
| 1 | | | 22 | | | 43 | | | 64 | | | 85 |
| --- | --- | --- | --- | --- | --- | --- | --- | --- | --- | --- | --- | --- |
Day
Capecitabine
2 weeks
2 weeks
2 weeks
2 weeks
Course 1
Course 2
Course 3
Course 4
Cystine/Theanine
or
Placebo
Four courses of capecitabine therapy in 12 weeks
Supplemental Fig 1. Schematic diagram of capecitabine and intervention treatment
