# Supplementary material for: Efficacy of oral administration of cystine and theanine in colorectal cancer patients undergoing capecitabine-based adjuvant chemotherapy after surgery: a multi-institutional, randomized, double-blinded, placebo-controlled, phase II trial (JORTC-CAM03)
Source: Support Care Cancer. 2019 Dec 6;28(8):3649–57. doi: 10.1007/s00520-019-05205-1 (PMC7316838; doi:10.1007/s00520-019-05205-1)
Supplement: Supplementary file 2 — (DOCX 202 kb). [file 520_2019_5205_MOESM2_ESM.docx]

**Supplemental Table 1. Hand-foot syndrome grading scale***

| Grade | Clinical domain** | Functional domain |
| --- | --- | --- |
| 1 | Numbness, dysesthesia/paresthesia, tingling, painless swelling, or erythema | Discomfort that does not disrupt normal activities |
| 2 | Painful erythema with swelling | Discomfort that affects activities of daily living |
| 3 | Moist desquamation, ulceration, blistering, and severe pain | Severe discomfort, unable to work or perform activities of daily living |
| *This scale is modified from [13] and applies only for the grading of hand-foot syndrome and not for any other skin abnormalities or other cutaneous areas.  **In the case of a discrepancy between clinical and functional domains, the assigned grade will be the higher of the grades. | | |

**Supplemental Table 2. Adherence to cystine/theanine or placebo treatment**

|  | Cystine/theanine (n = 49) | | | | |
| --- | --- | --- | --- | --- | --- |
|  | Course 1 | Course 2 | Course 3 | Course 4 | Total |
| Number of patients | 47 | 44 | 43 | 43 | 47 |
| Mean ± SD (%) | 98.91 ± 4.98 | 99.24 ± 5.03 | 99.67 ± 1.08 | 99.78 ± 1.45 | 99.41 ± 2.34 |
|  | Placebo (n = 45) | | | | |
|  | Course 1 | Course 2 | Course 3 | Course 4 | Total |
| Number of patients | 43 | 39 | 36 | 32 | 44 |
| Mean ± SD (%) | 96.84 ± 9.16 | 97.95 ± 6.44 | 98.69 ± 5.42 | 96.81 ± 8.97 | 97.47 ± 5.36 |

Abbreviations: SD, standard deviation

**Supplemental Table 3. Scores of EORTC QLQ-C30 and EORTC QLQ-CR29 (complete case analysis)**

|  |  |  | Cystine/theanine (n = 49) | | | Placebo (n = 45) | | |  |
| --- | --- | --- | --- | --- | --- | --- | --- | --- | --- |
| EORTC QLQ-C30 | |  | No. of patients | Mean ± SD | Median (range) | No. of patients | Mean ± SD | Median (range) | *p*-value |
| Global health status/QoL | Before | Course 1 | 46 | 67.9 ± 20.6 | 66.7 (17−100) | 44 | 71.8 ± 19.7 | 75.0 (17−100) | − |
|  |  | Course 2 | 44 | 69.5 ± 20.6 | 66.7 (33−100) | 39 | 70.7 ± 16.5 | 66.7 (33−100) | 0.7687 |
|  |  | Course 3 | 42 | 71.6 ± 19.0 | 79.2 (25−100) | 39 | 66.9 ± 22.9 | 66.7 (0−100) | 0.3122 |
|  |  | Course 4 | 43 | 72.9 ± 20.6 | 83.3 (17−100) | 38 | 69.7 ± 16.9 | 66.7 (33−100) | 0.4605 |
|  | After | Course 4 | 41 | 68.1 ± 22.4 | 66.7 (17−100) | 38 | 69.7 ± 19.1 | 66.7 (25−100) | 0.7273 |
| Physical functioning | Before | Course 1 | 49 | 91.9 ± 13.3 | 100.0 (27−100) | 44 | 88.4 ± 10.9 | 93.3 (67−100) | − |
|  |  | Course 2 | 45 | 93.5 ± 8.8 | 93.3 (53−100) | 40 | 89.0 ± 10.7 | 92.5 (60−100) | 0.0339 |
|  |  | Course 3 | 43 | 92.9 ± 7.9 | 93.3 (67−100) | 39 | 89.1 ± 13.6 | 93.3 (33−100) | 0.1209 |
|  |  | Course 4 | 43 | 92.5 ± 10.6 | 93.3 (47−100) | 37 | 91.4 ± 8.4 | 93.3 (67−100) | 0.5911 |
|  | After | Course 4 | 42 | 91.1 ± 12.1 | 93.3 (47−100) | 38 | 91.8 ± 8.3 | 93.3 (73−100) | 0.7714 |
| Role functioning | Before | Course 1 | 49 | 84.0 ± 22.3 | 100.0 (0−100) | 44 | 84.1 ± 19.3 | 100.0 (33−100) | − |
|  |  | Course 2 | 45 | 88.9 ± 16.3 | 100.0 (33−100) | 40 | 87.1 ± 15.8 | 100.0 (50−100) | 0.6061 |
|  |  | Course 3 | 43 | 85.7 ± 16.1 | 100.0 (67−100) | 39 | 85.0 ± 20.9 | 100.0 (0−100) | 0.8808 |
|  |  | Course 4 | 43 | 88.4 ± 16.9 | 100.0 (33−100) | 37 | 87.4 ± 14.9 | 100.0 (67−100) | 0.7845 |
|  | After | Course 4 | 42 | 86.5 ± 19.2 | 100.0 (33−100) | 38 | 85.5 ± 15.6 | 91.7 (67−100) | 0.8037 |
| Emotional functioning | Before | Course 1 | 47 | 83.2 ± 15.5 | 83.3 (42−100) | 44 | 87.3 ± 12.1 | 91.7 (58−100) | − |
|  |  | Course 2 | 45 | 88.7 ± 11.4 | 91.7 (67−100) | 39 | 88.7 ± 12.2 | 91.7 (67−100) | 0.9912 |
|  |  | Course 3 | 43 | 88.4 ± 11.5 | 91.7 (58−100) | 39 | 89.5 ± 12.4 | 91.7 (67−100) | 0.6617 |
|  |  | Course 4 | 44 | 89.2 ± 12.7 | 91.7 (42−100) | 38 | 91.0 ± 12.0 | 100.0 (58−100) | 0.5116 |
|  | After | Course 4 | 42 | 88.3 ± 11.7 | 91.7 (58−100) | 38 | 90.0 ± 12.2 | 100.0 (67−100) | 0.5277 |
| Cognitive functioning | Before | Course 1 | 47 | 91.5 ± 12.5 | 100.0 (67−100) | 44 | 89.8 ± 13.1 | 100.0 (50−100) | − |
|  |  | Course 2 | 45 | 93.7 ± 11.9 | 100.0 (50−100) | 39 | 88.9 ± 12.9 | 83.3 (50−100) | 0.0792 |
|  |  | Course 3 | 43 | 91.9 ± 14.3 | 100.0 (33−100) | 39 | 87.6 ± 13.1 | 83.3 (67−100) | 0.1646 |
|  |  | Course 4 | 44 | 90.2 ± 16.6 | 100.0 (33−100) | 38 | 85.5 ± 14.1 | 83.3 (67−100) | 0.1810 |
|  | After | Course 4 | 42 | 90.9 ± 15.7 | 100.0 (50−100) | 38 | 86.4 ± 13.9 | 83.3 (67−100) | 0.1834 |
| Social functioning | Before | Course 1 | 47 | 86.5 ± 17.6 | 100.0 (33−100) | 44 | 88.6 ± 15.6 | 100.0 (33−100) | − |
|  |  | Course 2 | 45 | 89.3 ± 14.3 | 100.0 (50−100) | 39 | 91.9 ± 12.0 | 100.0 (67−100) | 0.3696 |
|  |  | Course 3 | 43 | 92.6 ± 13.3 | 100.0 (50−100) | 39 | 90.2 ± 14.2 | 100.0 (50−100) | 0.4182 |
|  |  | Course 4 | 44 | 90.2 ± 17.4 | 100.0 (33−100) | 38 | 91.7 ± 12.1 | 100.0 (67−100) | 0.6529 |
|  | After | Course 4 | 42 | 90.1 ± 16.1 | 100.0 (33−100) | 38 | 90.4 ± 13.8 | 100.0 (67−100) | 0.9359 |
| Fatigue | Before | Course 1 | 48 | 23.8 ± 16.8 | 22.2 (0−67) | 44 | 22.5 ± 15.1 | 22.2 (0−44) | − |
|  |  | Course 2 | 45 | 21.7 ± 14.2 | 22.2 (0−44) | 40 | 21.5 ± 14.7 | 22.2 (0−44) | 0.9491 |
|  |  | Course 3 | 43 | 21.4 ± 16.5 | 22.2 (0−56) | 39 | 23.1 ± 15.4 | 22.2 (0−67) | 0.6460 |
|  |  | Course 4 | 43 | 19.4 ± 16.8 | 22.2 (0−56) | 37 | 20.1 ± 13.6 | 22.2 (0−44) | 0.8308 |
|  | After | Course 4 | 42 | 20.9 ± 15.8 | 22.2 (0−56) | 38 | 20.0 ± 14.0 | 22.2 (0−50) | 0.7955 |
| Nausea and vomiting | Before | Course 1 | 49 | 1.0 ± 4.0 | 0.0 (0−17) | 44 | 0.4 ± 2.5 | 0.0 (0−17) | − |
|  |  | Course 2 | 45 | 3.7 ± 9.3 | 0.0 (0−33) | 40 | 2.9 ± 7.4 | 0.0 (0−33) | 0.6709 |
|  |  | Course 3 | 43 | 2.3 ± 6.9 | 0.0 (0−33) | 39 | 3.0 ± 9.3 | 0.0 (0−50) | 0.7111 |
|  |  | Course 4 | 43 | 3.5 ± 7.8 | 0.0 (0−33) | 37 | 3.2 ± 7.7 | 0.0 (0−33) | 0.8472 |
|  | After | Course 4 | 42 | 2.4 ± 7.0 | 0.0 (0−33) | 38 | 5.3 ± 13.5 | 0.0 (0−67) | 0.2268 |
| Pain | Before | Course 1 | 49 | 15.3 ± 13.5 | 16.7 (0−33) | 44 | 16.3 ± 18.5 | 16.7 (0−67) | − |
|  |  | Course 2 | 46 | 9.8 ± 14.7 | 0.0 (0−50) | 40 | 16.3 ± 17.1 | 16.7 (0−67) | 0.0628 |
|  |  | Course 3 | 43 | 11.6 ± 13.9 | 0.0 (0−33) | 39 | 17.9 ± 21.1 | 16.7 (0−100) | 0.1095 |
|  |  | Course 4 | 44 | 11.0 ± 15.6 | 0.0 (0−67) | 38 | 13.2 ± 14.1 | 16.7 (0−33) | 0.5129 |
|  | After | Course 4 | 42 | 14.7 ± 21.8 | 0.0 (0−100) | 38 | 12.7 ± 17.1 | 0.0 (0−50) | 0.6579 |
| Dyspnea | Before | Course 1 | 49 | 7.5 ± 14.1 | 0.0 (0−33) | 44 | 6.1 ± 13.0 | 0.0 (0−33) | − |
|  |  | Course 2 | 44 | 6.8 ± 13.6 | 0.0 (0−33) | 40 | 9.2 ± 15.1 | 0.0 (0−33) | 0.4550 |
|  |  | Course 3 | 43 | 7.0 ± 13.7 | 0.0 (0−33) | 39 | 6.8 ± 13.6 | 0.0 (0−33) | 0.9634 |
|  |  | Course 4 | 43 | 6.2 ± 13.1 | 0.0 (0−33) | 37 | 8.1 ± 14.5 | 0.0 (0−33) | 0.5389 |
|  | After | Course 4 | 42 | 7.1 ± 13.8 | 0.0 (0−33) | 37 | 8.1 ± 14.5 | 0.0 (0−33) | 0.7631 |
| Insomnia | Before | Course 1 | 49 | 7.5 ± 15.6 | 0.0 (0−67) | 44 | 12.9 ± 16.4 | 0.0 (0−33) | − |
|  |  | Course 2 | 45 | 13.3 ± 18.0 | 0.0 (0−67) | 40 | 13.3 ± 18.2 | 0.0 (0−67) | 1.0000 |
|  |  | Course 3 | 43 | 10.1 ± 17.1 | 0.0 (0−67) | 39 | 15.4 ± 22.7 | 0.0 (0−100) | 0.2334 |
|  |  | Course 4 | 43 | 8.5 ± 14.7 | 0.0 (0−33) | 37 | 9.0 ± 15.0 | 0.0 (0−33) | 0.8853 |
|  | After | Course 4 | 42 | 7.1 ± 13.8 | 0.0 (0−33) | 38 | 12.3 ± 18.0 | 0.0 (0−67) | 0.1548 |
| Appetite loss | Before | Course 1 | 49 | 12.9 ± 17.8 | 0.0 (0−67) | 44 | 6.8 ± 13.6 | 0.0 (0−33) | − |
|  |  | Course 2 | 45 | 9.6 ± 15.3 | 0.0 (0−33) | 40 | 10.0 ± 17.2 | 0.0 (0−67) | 0.9165 |
|  |  | Course 3 | 43 | 8.5 ± 16.4 | 0.0 (0−67) | 39 | 12.0 ± 22.3 | 0.0 (0−100) | 0.4257 |
|  |  | Course 4 | 43 | 6.2 ± 15.0 | 0.0 (0−67) | 37 | 10.8 ± 19.3 | 0.0 (0−67) | 0.2340 |
|  | After | Course 4 | 42 | 8.7 ± 16.6 | 0.0 (0−67) | 38 | 7.9 ± 21.1 | 0.0 (0−100) | 0.8437 |
| Constipation | Before | Course 1 | 49 | 8.2 ± 16.0 | 0.0 (0−67) | 43 | 20.2 ± 23.2 | 33.3 (0−100) | − |
|  |  | Course 2 | 45 | 7.4 ± 14.0 | 0.0 (0−33) | 40 | 14.2 ± 23.7 | 0.0 (0−100) | 0.1091 |
|  |  | Course 3 | 43 | 5.4 ± 12.5 | 0.0 (0−33) | 39 | 13.7 ± 21.2 | 0.0 (0−67) | 0.0330 |
|  |  | Course 4 | 43 | 5.4 ± 12.5 | 0.0 (0−33) | 37 | 14.4 ± 23.0 | 0.0 (0−100) | 0.0295 |
|  | After | Course 4 | 42 | 4.8 ± 11.8 | 0.0 (0−33) | 38 | 15.8 ± 22.9 | 0.0 (0−100) | 0.0075 |
| Diarrhea | Before | Course 1 | 46 | 13.8 ± 21.8 | 0.0 (0−100) | 44 | 13.6 ± 19.5 | 0.0 (0−67) | − |
|  |  | Course 2 | 45 | 16.3 ± 20.9 | 0.0 (0−67) | 39 | 16.2 ± 18.5 | 0.0 (0−67) | 0.9895 |
|  |  | Course 3 | 43 | 13.2 ± 19.4 | 0.0 (0−67) | 39 | 17.1 ± 26.4 | 0.0 (0−100) | 0.4432 |
|  |  | Course 4 | 44 | 12.1 ± 19.1 | 0.0 (0−67) | 38 | 14.9 ± 16.8 | 0.0 (0−33) | 0.4881 |
|  | After | Course 4 | 42 | 9.5 ± 18.5 | 0.0 (0−67) | 38 | 14.0 ± 16.7 | 0.0 (0−33) | 0.2567 |
| Financial difficulties | Before | Course 1 | 47 | 12.8 ± 19.1 | 0.0 (0−67) | 44 | 12.9 ± 21.8 | 0.0 (0−100) | − |
|  |  | Course 2 | 45 | 14.8 ± 20.8 | 0.0 (0−67) | 39 | 11.1 ± 15.9 | 0.0 (0−33) | 0.3677 |
|  |  | Course 3 | 43 | 9.3 ± 16.8 | 0.0 (0−67) | 38 | 10.5 ± 22.1 | 0.0 (0−100) | 0.7780 |
|  |  | Course 4 | 44 | 10.6 ± 18.7 | 0.0 (0−67) | 38 | 8.8 ± 14.9 | 0.0 (0−33) | 0.6283 |
|  | After | Course 4 | 42 | 9.5 ± 16.9 | 0.0 (0−67) | 38 | 8.8 ± 14.9 | 0.0 (0−33) | 0.8342 |
| EORTC QLQ-CR29 | |  |  |  |  |  |  |  |  |
| Body image | Before | Course 1 | 49 | 81.4 ± 20.3 | 88.9 (0−100) | 44 | 84.8 ± 15.9 | 88.9 (33−100) | − |
|  |  | Course 2 | 45 | 84.4 ± 16.9 | 88.9 (33−100) | 39 | 85.6 ± 13.2 | 88.9 (67−100) | 0.7286 |
|  |  | Course 3 | 42 | 83.7 ± 17.5 | 88.9 (33−100) | 39 | 85.2 ± 15.2 | 88.9 (56−100) | 0.6916 |
|  |  | Course 4 | 44 | 82.8 ± 17.4 | 88.9 (33−100) | 38 | 85.4 ± 13.7 | 88.9 (56−100) | 0.4671 |
|  | After | Course 4 | 42 | 83.9 ± 16.3 | 88.9 (33−100) | 38 | 84.2 ± 15.6 | 88.9 (33−100) | 0.9228 |
| Anxiety | Before | Course 1 | 49 | 69.4 ± 19.1 | 66.7 (33−100) | 44 | 73.5 ± 21.1 | 66.7 (33−100) | − |
|  |  | Course 2 | 45 | 71.1 ± 18.3 | 66.7 (0−100) | 39 | 78.6 ± 19.5 | 66.7 (33−100) | 0.0716 |
|  |  | Course 3 | 42 | 73.0 ± 15.2 | 66.7 (33−100) | 39 | 82.1 ± 18.5 | 66.7 (33−100) | 0.0182 |
|  |  | Course 4 | 44 | 74.2 ± 21.4 | 66.7 (0−100) | 38 | 78.9 ± 19.6 | 66.7 (33−100) | 0.3056 |
|  | After | Course 4 | 42 | 76.2 ± 18.5 | 66.7 (33−100) | 38 | 80.7 ± 20.0 | 66.7 (33−100) | 0.2968 |
| Weight | Before | Course 1 | 49 | 84.4 ± 25.6 | 100.0 (0−100) | 44 | 88.6 ± 16.0 | 100.0 (67−100) | − |
|  |  | Course 2 | 45 | 86.7 ± 19.3 | 100.0 (33−100) | 39 | 88.0 ± 16.2 | 100.0 (67−100) | 0.7285 |
|  |  | Course 3 | 42 | 86.5 ± 19.6 | 100.0 (33−100) | 39 | 88.0 ± 17.9 | 100.0 (33−100) | 0.7158 |
|  |  | Course 4 | 44 | 84.8 ± 19.6 | 100.0 (33−100) | 38 | 89.5 ± 15.7 | 100.0 (67−100) | 0.2473 |
|  | After | Course 4 | 42 | 84.1 ± 18.4 | 100.0 (33−100) | 38 | 88.6 ± 16.0 | 100.0 (67−100) | 0.2523 |
| Sexual interest (men) | Before | Course 1 | 25 | 77.3 ± 20.9 | 66.7 (33−100) | 23 | 79.7 ± 16.6 | 66.7 (67−100) | − |
|  |  | Course 2 | 29 | 79.3 ± 20.7 | 66.7 (33−100) | 19 | 77.2 ± 15.9 | 66.7 (67−100) | 0.7074 |
|  |  | Course 3 | 25 | 80.0 ± 19.3 | 66.7 (33−100) | 20 | 83.3 ± 17.1 | 83.3 (67−100) | 0.5475 |
|  |  | Course 4 | 26 | 80.8 ± 16.8 | 66.7 (67−100) | 19 | 84.2 ± 17.1 | 100.0 (67−100) | 0.5041 |
|  | After | Course 4 | 26 | 79.5 ± 19.0 | 66.7 (33−100) | 19 | 78.9 ± 19.9 | 66.7 (33−100) | 0.9270 |
| Sexual interest (women) | Before | Course 1 | 15 | 100.0 ± 0.0 | 100.0 (100−100) | 17 | 98.0 ± 8.1 | 100.0 (67−100) | − |
|  |  | Course 2 | 14 | 100.0 ± 0.0 | 100.0 (100−100) | 14 | 97.6 ± 8.9 | 100.0 (67−100) | 0.3265 |
|  |  | Course 3 | 11 | 97.0 ± 10.1 | 100.0 (67−100) | 14 | 100.0 ± 0.0 | 100.0 (100−100) | 0.2681 |
|  |  | Course 4 | 12 | 100.0 ± 0.0 | 100.0 (100−100) | 15 | 97.8 ± 8.6 | 100.0 (67−100) | 0.3815 |
|  | After | Course 4 | 11 | 100.0 ± 0.0 | 100.0 (100−100) | 14 | 97.6 ± 8.9 | 100.0 (67−100) | 0.3867 |
| Urinary frequency | Before | Course 1 | 48 | 22.9 ± 21.9 | 16.7 (0−100) | 44 | 23.1 ± 22.5 | 16.7 (0−67) | − |
|  |  | Course 2 | 44 | 20.8 ± 23.6 | 16.7 (0−100) | 38 | 25.4 ± 19.3 | 33.3 (0−67) | 0.3410 |
|  |  | Course 3 | 42 | 20.6 ± 19.1 | 16.7 (0−67) | 39 | 19.2 ± 19.7 | 16.7 (0−67) | 0.7453 |
|  |  | Course 4 | 43 | 18.6 ± 19.3 | 16.7 (0−67) | 38 | 15.4 ± 17.1 | 16.7 (0−50) | 0.4268 |
|  | After | Course 4 | 42 | 18.7 ± 17.0 | 16.7 (0−67) | 38 | 18.9 ± 17.4 | 16.7 (0−67) | 0.9568 |
| Blood and mucus in stool | Before | Course 1 | 49 | 5.1 ± 9.7 | 0.0 (0−33) | 44 | 3.0 ± 7.4 | 0.0 (0−33) | − |
|  |  | Course 2 | 45 | 3.0 ± 8.2 | 0.0 (0−33) | 39 | 2.6 ± 8.2 | 0.0 (0−33) | 0.8238 |
|  |  | Course 3 | 42 | 2.0 ± 6.6 | 0.0 (0−33) | 39 | 2.1 ± 5.6 | 0.0 (0−17) | 0.9115 |
|  |  | Course 4 | 44 | 2.7 ± 8.8 | 0.0 (0−50) | 38 | 2.2 ± 5.7 | 0.0 (0−17) | 0.7834 |
|  | After | Course 4 | 42 | 2.0 ± 6.6 | 0.0 (0−33) | 38 | 1.3 ± 4.6 | 0.0 (0−17) | 0.6028 |
| Stool frequency | Before | Course 1 | 38 | 17.1 ± 24.7 | 8.3 (0−100) | 40 | 20.0 ± 20.7 | 16.7 (0−67) | − |
|  |  | Course 2 | 38 | 16.7 ± 20.5 | 16.7 (0−67) | 35 | 19.0 ± 20.3 | 16.7 (0−67) | 0.6198 |
|  |  | Course 3 | 36 | 14.4 ± 15.5 | 16.7 (0−67) | 33 | 16.2 ± 18.4 | 16.7 (0−50) | 0.6591 |
|  |  | Course 4 | 36 | 12.5 ± 12.8 | 16.7 (0−50) | 35 | 16.7 ± 17.2 | 16.7 (0−67) | 0.2495 |
|  | After | Course 4 | 33 | 12.6 ± 14.5 | 16.7 (0−50) | 33 | 13.6 ± 16.9 | 16.7 (0−67) | 0.7950 |
| Urinary incontinence | Before | Course 1 | 49 | 4.1 ± 11.0 | 0.0 (0−33) | 43 | 7.0 ± 15.5 | 0.0 (0−67) | − |
|  |  | Course 2 | 45 | 6.7 ± 13.5 | 0.0 (0−33) | 39 | 3.4 ± 10.3 | 0.0 (0−33) | 0.2230 |
|  |  | Course 3 | 41 | 8.1 ± 14.5 | 0.0 (0−33) | 39 | 4.3 ± 13.6 | 0.0 (0−67) | 0.2245 |
|  |  | Course 4 | 44 | 6.8 ± 13.6 | 0.0 (0−33) | 38 | 5.3 ± 12.3 | 0.0 (0−33) | 0.5913 |
|  | After | Course 4 | 42 | 7.1 ± 13.8 | 0.0 (0−33) | 38 | 7.9 ± 14.4 | 0.0 (0−33) | 0.8123 |
| Dysuria | Before | Course 1 | 49 | 2.0 ± 8.1 | 0.0 (0−33) | 44 | 6.8 ± 17.0 | 0.0 (0−67) | − |
|  |  | Course 2 | 45 | 2.2 ± 8.4 | 0.0 (0−33) | 39 | 3.4 ± 10.3 | 0.0 (0−33) | 0.5583 |
|  |  | Course 3 | 42 | 0.8 ± 5.1 | 0.0 (0−33) | 39 | 6.0 ± 13.0 | 0.0 (0−33) | 0.0187 |
|  |  | Course 4 | 44 | 0.0 ± 0.0 | 0.0 (0−0) | 38 | 6.1 ± 13.1 | 0.0 (0−33) | 0.0026 |
|  | After | Course 4 | 41 | 0.0 ± 0.0 | 0.0 (0−0) | 38 | 0.9 ± 5.4 | 0.0 (0−33) | 0.3019 |
| Abdominal pain | Before | Course 1 | 49 | 12.9 ± 19.0 | 0.0 (0−67) | 43 | 12.4 ± 17.9 | 0.0 (0−67) | − |
|  |  | Course 2 | 44 | 9.1 ± 15.0 | 0.0 (0−33) | 39 | 12.8 ± 22.5 | 0.0 (0−100) | 0.3715 |
|  |  | Course 3 | 42 | 4.0 ± 10.9 | 0.0 (0−33) | 39 | 13.7 ± 21.2 | 0.0 (0−100) | 0.0107 |
|  |  | Course 4 | 44 | 8.3 ± 16.3 | 0.0 (0−67) | 38 | 7.0 ± 13.8 | 0.0 (0−33) | 0.6963 |
|  | After | Course 4 | 42 | 6.3 ± 13.3 | 0.0 (0−33) | 38 | 8.8 ± 14.9 | 0.0 (0−33) | 0.4433 |
| Buttock pain | Before | Course 1 | 48 | 9.0 ± 16.5 | 0.0 (0−67) | 43 | 13.2 ± 19.4 | 0.0 (0−67) | − |
|  |  | Course 2 | 45 | 8.9 ± 14.9 | 0.0 (0−33) | 39 | 13.7 ± 18.3 | 0.0 (0−67) | 0.1901 |
|  |  | Course 3 | 41 | 7.3 ± 15.8 | 0.0 (0−67) | 39 | 12.8 ± 18.1 | 0.0 (0−67) | 0.1515 |
|  |  | Course 4 | 44 | 7.6 ± 15.9 | 0.0 (0−67) | 38 | 10.5 ± 15.7 | 0.0 (0−33) | 0.4011 |
|  | After | Course 4 | 42 | 4.8 ± 11.8 | 0.0 (0−33) | 38 | 12.3 ± 16.3 | 0.0 (0−33) | 0.0198 |
| Bloating | Before | Course 1 | 49 | 19.0 ± 20.4 | 33.3 (0−67) | 43 | 16.3 ± 22.3 | 0.0 (0−100) | − |
|  |  | Course 2 | 44 | 12.9 ± 17.9 | 0.0 (0−67) | 39 | 16.2 ± 22.8 | 0.0 (0−100) | 0.4548 |
|  |  | Course 3 | 42 | 11.1 ± 17.5 | 0.0 (0−67) | 39 | 14.5 ± 18.4 | 0.0 (0−67) | 0.3945 |
|  |  | Course 4 | 44 | 9.1 ± 16.7 | 0.0 (0−67) | 38 | 9.6 ± 17.2 | 0.0 (0−67) | 0.8818 |
|  | After | Course 4 | 42 | 10.3 ± 17.3 | 0.0 (0−67) | 38 | 8.8 ± 14.9 | 0.0 (0−33) | 0.6705 |
| Dry mouth | Before | Course 1 | 49 | 15.0 ± 18.1 | 0.0 (0−67) | 44 | 6.8 ± 13.6 | 0.0 (0−33) | − |
|  |  | Course 2 | 45 | 18.5 ± 22.0 | 0.0 (0−67) | 39 | 15.4 ± 16.8 | 0.0 (0−33) | 0.4705 |
|  |  | Course 3 | 42 | 19.0 ± 18.3 | 33.3 (0−67) | 39 | 14.5 ± 18.4 | 0.0 (0−67) | 0.2709 |
|  |  | Course 4 | 44 | 12.9 ± 16.4 | 0.0 (0−33) | 38 | 14.9 ± 18.5 | 0.0 (0−67) | 0.5994 |
|  | After | Course 4 | 42 | 12.7 ± 16.4 | 0.0 (0−33) | 38 | 14.0 ± 16.7 | 0.0 (0−33) | 0.7188 |
| Hair loss | Before | Course 1 | 48 | 1.4 ± 6.7 | 0.0 (0−33) | 43 | 0.0 ± 0.0 | 0.0 (0−0) | − |
|  |  | Course 2 | 44 | 3.0 ± 9.7 | 0.0 (0−33) | 39 | 4.3 ± 11.3 | 0.0 (0−33) | 0.5908 |
|  |  | Course 3 | 42 | 2.4 ± 8.7 | 0.0 (0−33) | 39 | 4.3 ± 13.6 | 0.0 (0−67) | 0.4552 |
|  |  | Course 4 | 43 | 5.4 ± 12.5 | 0.0 (0−33) | 38 | 4.4 ± 11.4 | 0.0 (0−33) | 0.6975 |
|  | After | Course 4 | 41 | 5.7 ± 12.7 | 0.0 (0−33) | 38 | 6.1 ± 15.2 | 0.0 (0−67) | 0.8868 |
| Taste | Before | Course 1 | 49 | 3.4 ± 10.2 | 0.0 (0−33) | 44 | 3.0 ± 12.1 | 0.0 (0−67) | − |
|  |  | Course 2 | 44 | 8.3 ± 16.3 | 0.0 (0−67) | 39 | 6.0 ± 13.0 | 0.0 (0−33) | 0.4727 |
|  |  | Course 3 | 42 | 8.7 ± 16.6 | 0.0 (0−67) | 39 | 11.1 ± 20.7 | 0.0 (0−67) | 0.5680 |
|  |  | Course 4 | 44 | 12.9 ± 21.8 | 0.0 (0−67) | 38 | 10.5 ± 22.1 | 0.0 (0−100) | 0.6295 |
|  | After | Course 4 | 41 | 17.1 ± 22.5 | 0.0 (0−67) | 38 | 15.8 ± 24.2 | 0.0 (0−100) | 0.8076 |
| Flatulence | Before | Course 1 | 38 | 14.9 ± 16.8 | 0.0 (0−33) | 40 | 26.7 ± 26.4 | 33.3 (0−100) | − |
|  |  | Course 2 | 38 | 14.9 ± 18.5 | 0.0 (0−67) | 34 | 23.5 ± 19.3 | 33.3 (0−67) | 0.0572 |
|  |  | Course 3 | 36 | 14.8 ± 16.8 | 0.0 (0−33) | 33 | 22.2 ± 21.5 | 33.3 (0−67) | 0.1141 |
|  |  | Course 4 | 36 | 15.7 ± 18.7 | 0.0 (0−67) | 35 | 23.8 ± 22.3 | 33.3 (0−67) | 0.1020 |
|  | After | Course 4 | 33 | 16.2 ± 16.9 | 0.0 (0−33) | 32 | 18.8 ± 16.8 | 33.3 (0−33) | 0.5383 |
| Fecal incontinence | Before | Course 1 | 38 | 6.1 ± 15.2 | 0.0 (0−67) | 40 | 9.2 ± 22.6 | 0.0 (0−100) | − |
|  |  | Course 2 | 38 | 6.1 ± 13.1 | 0.0 (0−33) | 35 | 4.8 ± 11.8 | 0.0 (0−33) | 0.6395 |
|  |  | Course 3 | 36 | 6.5 ± 13.4 | 0.0 (0−33) | 32 | 8.3 ± 14.7 | 0.0 (0−33) | 0.5879 |
|  |  | Course 4 | 36 | 7.4 ± 14.1 | 0.0 (0−33) | 35 | 6.7 ± 13.5 | 0.0 (0−33) | 0.8217 |
|  | After | Course 4 | 33 | 7.1 ± 13.8 | 0.0 (0−33) | 32 | 4.2 ± 11.2 | 0.0 (0−33) | 0.3568 |
| Sore skin | Before | Course 1 | 38 | 11.4 ± 20.9 | 0.0 (0−67) | 40 | 17.5 ± 25.0 | 0.0 (0−100) | − |
|  |  | Course 2 | 38 | 10.5 ± 17.5 | 0.0 (0−67) | 35 | 18.1 ± 23.4 | 0.0 (0−67) | 0.1198 |
|  |  | Course 3 | 35 | 11.4 ± 18.0 | 0.0 (0−67) | 33 | 15.2 ± 22.2 | 0.0 (0−67) | 0.4486 |
|  |  | Course 4 | 36 | 8.3 ± 16.7 | 0.0 (0−67) | 35 | 13.3 ± 18.4 | 0.0 (0−67) | 0.2344 |
|  | After | Course 4 | 33 | 7.1 ± 16.2 | 0.0 (0−67) | 33 | 12.1 ± 16.3 | 0.0 (0−33) | 0.2105 |
| Embarrassment | Before | Course 1 | 38 | 1.8 ± 7.5 | 0.0 (0−33) | 40 | 3.3 ± 10.1 | 0.0 (0−33) | − |
|  |  | Course 2 | 38 | 1.8 ± 7.5 | 0.0 (0−33) | 35 | 2.9 ± 9.5 | 0.0 (0−33) | 0.5823 |
|  |  | Course 3 | 36 | 3.7 ± 10.6 | 0.0 (0−33) | 33 | 4.0 ± 11.1 | 0.0 (0−33) | 0.8977 |
|  |  | Course 4 | 36 | 2.8 ± 9.3 | 0.0 (0−33) | 35 | 4.8 ± 11.8 | 0.0 (0−33) | 0.4350 |
|  | After | Course 4 | 33 | 5.1 ± 12.1 | 0.0 (0−33) | 33 | 5.1 ± 12.1 | 0.0 (0−33) | 1.0000 |
| Stoma care problems | Before | Course 1 | 2 | 16.7 ± 23.6 | 16.7 (0−33) | 2 | 33.3 ± 0.0 | 33.3 (33−33) | − |
|  |  | Course 2 | 2 | 16.7 ± 23.6 | 16.7 (0−33) | 2 | 16.7 ± 23.6 | 16.7 (0−33) | 1.0000 |
|  |  | Course 3 | 2 | 16.7 ± 23.6 | 16.7 (0−33) | 2 | 33.3 ± 0.0 | 33.3 (33−33) | 0.4226 |
|  |  | Course 4 | 2 | 16.7 ± 23.6 | 16.7 (0−33) | 2 | 16.7 ± 23.6 | 16.7 (0−33) | 1.0000 |
|  | After | Course 4 | 2 | 16.7 ± 23.6 | 16.7 (0−33) | 2 | 33.3 ± 0.0 | 33.3 (33−33) | 0.4226 |
| Impotence | Before | Course 1 | 25 | 14.7 ± 21.7 | 0.0 (0−67) | 22 | 27.3 ± 33.6 | 16.7 (0−100) | − |
|  |  | Course 2 | 27 | 19.8 ± 23.1 | 0.0 (0−67) | 18 | 24.1 ± 27.6 | 33.3 (0−100) | 0.5725 |
|  |  | Course 3 | 25 | 18.7 ± 23.7 | 0.0 (0−67) | 19 | 24.6 ± 31.1 | 0.0 (0−100) | 0.4794 |
|  |  | Course 4 | 25 | 18.7 ± 25.6 | 0.0 (0−100) | 18 | 16.7 ± 23.6 | 0.0 (0−67) | 0.7953 |
|  | After | Course 4 | 25 | 14.7 ± 19.4 | 0.0 (0−67) | 19 | 22.8 ± 35.2 | 0.0 (0−100) | 0.3336 |
| Dyspareunia | Before | Course 1 | 12 | 0.0 ± 0.0 | 0.0 (0−0) | 11 | 6.1 ± 13.5 | 0.0 (0−33) | ‒ |
|  |  | Course 2 | 10 | 0.0 ± 0.0 | 0.0 (0−0) | 12 | 2.8 ± 9.6 | 0.0 (0−33) | 0.3741 |
|  |  | Course 3 | 11 | 6.1 ± 13.5 | 0.0 (0−33) | 10 | 3.3 ± 10.5 | 0.0 (0−33) | 0.6142 |
|  |  | Course 4 | 11 | 0.0 ± 0.0 | 0.0 (0−0) | 12 | 0.0 ± 0.0 | 0.0 (0−0) | NA |
|  | After | Course 4 | 11 | 6.1 ± 13.5 | 0.0 (0−33) | 9 | 0.0 ± 0.0 | 0.0 (0−0) | 0.1964 |

Abbreviations: EORTC QLQ-C30 and QLQ-CR29, European Organisation for Research and Treatment of Cancer Quality of Life Questionnaire module for all cancer patients (QLQ-C30) and for colorectal cancer patients (QLQ-CR29); SD, standard deviation; QoL, Quality of Life

**Supplemental Table 4. Scores of EORTC QLQ-C30 and EORTC QLQ-CR29 (last observation carried forward analysis)**

|  |  |  | Cystine/theanine (n = 49) | | | Placebo (n = 45) | | |  |
| --- | --- | --- | --- | --- | --- | --- | --- | --- | --- |
| EORTC QLQ-C30 | |  | No. of patients | Mean ± SD | Median (range) | No. of patients | Mean ± SD | Median (range) | *p*-value |
| Global health status/QoL | Before | Course 1 | 46 | 67.9 ± 20.6 | 66.7 (17−100) | 44 | 71.8 ± 19.7 | 75.0 (17−100) | − |
|  |  | Course 2 | 48 | 70.3 ± 20.5 | 66.7 (33−100) | 44 | 70.5 ± 16.6 | 66.7 (33−100) | 0.9711 |
|  |  | Course 3 | 48 | 72.7 ± 19.0 | 79.2 (25−100) | 44 | 67.0 ± 22.3 | 66.7 (0−100) | 0.1898 |
|  |  | Course 4 | 48 | 73.3 ± 20.2 | 83.3 (17−100) | 44 | 68.0 ± 19.8 | 66.7 (0−100) | 0.2096 |
|  | After | Course 4 | 48 | 69.3 ± 22.2 | 66.7 (17−100) | 44 | 68.0 ± 21.4 | 66.7 (0−100) | 0.7794 |
| Physical functioning | Before | Course 1 | 49 | 91.9 ± 13.3 | 100.0 (27−100) | 44 | 88.4 ± 10.9 | 93.3 (67−100) | − |
|  |  | Course 2 | 49 | 93.9 ± 8.6 | 93.3 (53−100) | 44 | 88.8 ± 10.7 | 92.5 (60−100) | 0.0115 |
|  |  | Course 3 | 49 | 93.5 ± 7.6 | 93.3 (67−100) | 44 | 89.1 ± 13.3 | 93.3 (33−100) | 0.0520 |
|  |  | Course 4 | 49 | 92.8 ± 10.2 | 93.3 (47−100) | 44 | 89.8 ± 12.2 | 93.3 (33−100) | 0.2039 |
|  | After | Course 4 | 49 | 91.8 ± 11.5 | 93.3 (47−100) | 44 | 90.2 ± 12.2 | 93.3 (33−100) | 0.5039 |
| Role functioning | Before | Course 1 | 49 | 84.0 ± 22.3 | 100.0 (0−100) | 44 | 84.1 ± 19.3 | 100.0 (33−100) | − |
|  |  | Course 2 | 49 | 89.8 ± 15.9 | 100.0 (33−100) | 44 | 87.5 ± 15.7 | 100.0 (50−100) | 0.4862 |
|  |  | Course 3 | 49 | 87.4 ± 15.8 | 100.0 (67−100) | 44 | 86.0 ± 20.3 | 100.0 (0−100) | 0.7040 |
|  |  | Course 4 | 49 | 89.1 ± 16.5 | 100.0 (33−100) | 44 | 86.4 ± 19.8 | 100.0 (0−100) | 0.4667 |
|  | After | Course 4 | 49 | 87.8 ± 18.6 | 100.0 (33−100) | 44 | 84.5 ± 20.1 | 100.0 (0−100) | 0.4152 |
| Emotional functioning | Before | Course 1 | 47 | 83.2 ± 15.5 | 83.3 (42−100) | 44 | 87.3 ± 12.1 | 91.7 (58−100) | − |
|  |  | Course 2 | 49 | 88.8 ± 11.1 | 91.7 (67−100) | 44 | 89.2 ± 12.0 | 91.7 (67−100) | 0.8582 |
|  |  | Course 3 | 49 | 88.8 ± 11.1 | 91.7 (58−100) | 44 | 90.0 ± 12.1 | 91.7 (67−100) | 0.6235 |
|  |  | Course 4 | 49 | 89.3 ± 12.2 | 91.7 (42−100) | 44 | 90.7 ± 12.2 | 100.0 (58−100) | 0.5722 |
|  | After | Course 4 | 49 | 88.4 ± 11.3 | 91.7 (58−100) | 44 | 89.8 ± 12.4 | 100.0 (67−100) | 0.5689 |
| Cognitive functioning | Before | Course 1 | 47 | 91.5 ± 12.5 | 100.0 (67−100) | 44 | 89.8 ± 13.1 | 100.0 (50−100) | − |
|  |  | Course 2 | 49 | 92.9 ± 12.7 | 100.0 (50−100) | 44 | 89.0 ± 12.4 | 83.3 (50−100) | 0.1450 |
|  |  | Course 3 | 49 | 91.5 ± 14.5 | 100.0 (33−100) | 44 | 88.3 ± 12.8 | 83.3 (67−100) | 0.2576 |
|  |  | Course 4 | 49 | 89.8 ± 16.6 | 100.0 (33−100) | 44 | 86.4 ± 13.6 | 83.3 (67−100) | 0.2813 |
|  | After | Course 4 | 49 | 90.1 ± 15.9 | 100.0 (50−100) | 44 | 87.1 ± 13.4 | 83.3 (67−100) | 0.3286 |
| Social functioning | Before | Course 1 | 47 | 86.5 ± 17.6 | 100.0 (33−100) | 44 | 88.6 ± 15.6 | 100.0 (33−100) | − |
|  |  | Course 2 | 49 | 89.5 ± 13.9 | 100.0 (50−100) | 44 | 92.0 ± 12.2 | 100.0 (67−100) | 0.3443 |
|  |  | Course 3 | 49 | 92.9 ± 12.7 | 100.0 (50−100) | 44 | 91.3 ± 13.7 | 100.0 (50−100) | 0.5681 |
|  |  | Course 4 | 49 | 90.5 ± 16.7 | 100.0 (33−100) | 44 | 91.7 ± 13.2 | 100.0 (50−100) | 0.7057 |
|  | After | Course 4 | 49 | 90.5 ± 15.2 | 100.0 (33−100) | 44 | 90.5 ± 14.6 | 100.0 (50−100) | 0.9861 |
| Fatigue | Before | Course 1 | 48 | 23.8 ± 16.8 | 22.2 (0−67) | 44 | 22.5 ± 15.1 | 22.2 (0−44) | − |
|  |  | Course 2 | 49 | 21.5 ± 14.1 | 22.2 (0−44) | 44 | 21.6 ± 15.0 | 22.2 (0−44) | 0.9871 |
|  |  | Course 3 | 49 | 20.6 ± 16.5 | 22.2 (0−56) | 44 | 23.5 ± 15.2 | 22.2 (0−67) | 0.3905 |
|  |  | Course 4 | 49 | 19.3 ± 16.5 | 22.2 (0−56) | 44 | 22.0 ± 15.1 | 22.2 (0−67) | 0.4142 |
|  | After | Course 4 | 49 | 20.4 ± 15.8 | 22.2 (0−56) | 44 | 21.8 ± 15.6 | 22.2 (0−67) | 0.6602 |
| Nausea and vomiting | Before | Course 1 | 49 | 1.0 ± 4.0 | 0.0 (0−17) | 44 | 0.4 ± 2.51 | 0.0 (0−17) | − |
|  |  | Course 2 | 49 | 3.4 ± 9.0 | 0.0 (0−33) | 44 | 2.7 ± 7.1 | 0.0 (0−33) | 0.6594 |
|  |  | Course 3 | 49 | 2.4 ± 6.8 | 0.0 (0−33) | 44 | 2.7 ± 8.8 | 0.0 (0−50) | 0.8676 |
|  |  | Course 4 | 49 | 3.4 ± 7.6 | 0.0 (0−33) | 44 | 3.8 ± 10.1 | 0.0 (0−50) | 0.8340 |
|  | After | Course 4 | 49 | 2.7 ± 7.1 | 0.0 (0−33) | 44 | 5.7 ± 14.4 | 0.0 (0−67) | 0.2035 |
| Pain | Before | Course 1 | 49 | 15.3 ± 13.5 | 16.7 (0−33) | 44 | 16.3 ± 18.5 | 16.7 (0−67) | − |
|  |  | Course 2 | 49 | 10.2 ± 14.8 | 0.0 (0−50) | 44 | 15.5 ± 17.0 | 16.7 (0−67) | 0.1094 |
|  |  | Course 3 | 49 | 11.9 ± 14.0 | 0.0 (0−33) | 44 | 17.4 ± 20.6 | 16.7 (0−100) | 0.1315 |
|  |  | Course 4 | 49 | 11.6 ± 15.7 | 0.0 (0−67) | 44 | 15.2 ± 19.3 | 16.7 (0−100) | 0.3256 |
|  | After | Course 4 | 49 | 14.6 ± 20.9 | 0.0 (0−100) | 44 | 14.8 ± 21.3 | 0.0 (0−100) | 0.9733 |
| Dyspnea | Before | Course 1 | 49 | 7.5 ± 14.1 | 0.0 (0−33) | 44 | 6.1 ± 13.0 | 0.0 (0−33) | − |
|  |  | Course 2 | 49 | 6.1 ± 13.0 | 0.0 (0−33) | 44 | 8.3 ± 14.6 | 0.0 (0−33) | 0.4425 |
|  |  | Course 3 | 49 | 6.1 ± 13.0 | 0.0 (0−33) | 44 | 6.1 ± 13.0 | 0.0 (0−33) | 0.9818 |
|  |  | Course 4 | 49 | 5.4 ± 12.5 | 0.0 (0−33) | 44 | 6.8 ± 13.6 | 0.0 (0−33) | 0.6117 |
|  | After | Course 4 | 49 | 6.8 ± 13.6 | 0.0 (0−33) | 44 | 6.8 ± 13.6 | 0.0 (0−33) | 0.9956 |
| Insomnia | Before | Course 1 | 49 | 7.5 ± 15.6 | 0.0 (0−67) | 44 | 12.9 ± 16.4 | 0.0 (0−33) | − |
|  |  | Course 2 | 49 | 12.2 ± 17.6 | 0.0 (0−67) | 44 | 12.9 ± 17.9 | 0.0 (0−67) | 0.8639 |
|  |  | Course 3 | 49 | 9.5 ± 16.7 | 0.0 (0−67) | 44 | 15.2 ± 22.1 | 0.0 (0−100) | 0.1665 |
|  |  | Course 4 | 49 | 8.2 ± 14.5 | 0.0 (0−33) | 44 | 11.4 ± 20.3 | 0.0 (0−100) | 0.3796 |
|  | After | Course 4 | 49 | 6.8 ± 13.6 | 0.0 (0−33) | 44 | 14.4 ± 22.0 | 0.0 (0−100) | 0.0461 |
| Appetite loss | Before | Course 1 | 49 | 12.9 ± 17.8 | 0.0 (0−67) | 44 | 6.8 ± 13.6 | 0.0 (0−33) | − |
|  |  | Course 2 | 49 | 9.5 ± 15.2 | 0.0 (0−33) | 44 | 9.8 ± 17.0 | 0.0 (0−67) | 0.9227 |
|  |  | Course 3 | 49 | 8.2 ± 16.0 | 0.0 (0−67) | 44 | 11.4 ± 21.5 | 0.0 (0−100) | 0.4146 |
|  |  | Course 4 | 49 | 6.1 ± 14.7 | 0.0 (0−67) | 44 | 12.1 ± 22.8 | 0.0 (0−100) | 0.1317 |
|  | After | Course 4 | 49 | 8.2 ± 16.0 | 0.0 (0−67) | 44 | 9.8 ± 24.5 | 0.0 (0−100) | 0.6923 |
| Constipation | Before | Course 1 | 49 | 8.2 ± 16.0 | 0.0 (0−67) | 43 | 20.2 ± 23.2 | 33.3 (0−100) | − |
|  |  | Course 2 | 49 | 7.5 ± 14.1 | 0.0 (0−33) | 44 | 15.2 ± 24.3 | 0.0 (0−100) | 0.0627 |
|  |  | Course 3 | 49 | 5.4 ± 12.5 | 0.0 (0−33) | 44 | 14.4 ± 22.0 | 0.0 (0−67) | 0.0165 |
|  |  | Course 4 | 49 | 5.4 ± 12.5 | 0.0 (0−33) | 44 | 15.2 ± 23.2 | 0.0 (0−100) | 0.0126 |
|  | After | Course 4 | 49 | 5.4 ± 12.5 | 0.0 (0−33) | 44 | 16.7 ± 23.3 | 0.0 (0−100) | 0.0042 |
| Diarrhea | Before | Course 1 | 46 | 13.8 ± 21.8 | 0.0 (0−100) | 44 | 13.6 ± 19.5 | 0.0 (0−67) | − |
|  |  | Course 2 | 49 | 15.6 ± 20.5 | 0.0 (0−67) | 44 | 15.2 ± 18.3 | 0.0 (0−67) | 0.9030 |
|  |  | Course 3 | 49 | 12.2 ± 18.9 | 0.0 (0−67) | 44 | 15.9 ± 25.4 | 0.0 (0−100) | 0.4289 |
|  |  | Course 4 | 49 | 11.6 ± 18.7 | 0.0 (0−67) | 44 | 15.9 ± 20.9 | 0.0 (0−100) | 0.2933 |
|  | After | Course 4 | 49 | 9.5 ± 18.0 | 0.0 (0−67) | 44 | 15.2 ± 20.9 | 0.0 (0−100) | 0.1665 |
| Financial difficulties | Before | Course 1 | 47 | 12.8 ± 19.1 | 0.0 (0−67) | 44 | 12.9 ± 21.8 | 0.0 (0−100) | − |
|  |  | Course 2 | 49 | 14.3 ± 20.4 | 0.0 (0−67) | 44 | 10.6 ± 15.7 | 0.0 (0−33) | 0.3366 |
|  |  | Course 3 | 49 | 8.8 ± 16.4 | 0.0 (0−67) | 44 | 9.1 ± 20.8 | 0.0 (0−100) | 0.9490 |
|  |  | Course 4 | 49 | 10.2 ± 18.3 | 0.0 (0−67) | 44 | 9.8 ± 19.8 | 0.0 (0−100) | 0.9284 |
|  | After | Course 4 | 49 | 9.5 ± 16.7 | 0.0 (0−67) | 44 | 9.8 ± 19.8 | 0.0 (0−100) | 0.9318 |
| EORTC QLQ-CR29 | |  |  |  |  |  |  |  |  |
| Body image | Before | Course 1 | 49 | 81.4 ± 20.3 | 88.9 (0−100) | 44 | 84.8 ± 15.9 | 88.9 (33−100) | − |
|  |  | Course 2 | 49 | 84.8 ± 16.7 | 88.9 (33−100) | 44 | 84.5 ± 14.4 | 88.9 (44−100) | 0.9177 |
|  |  | Course 3 | 49 | 85.4 ± 16.9 | 88.9 (33−100) | 44 | 84.3 ± 15.8 | 88.9 (44−100) | 0.7621 |
|  |  | Course 4 | 49 | 84.1 ± 17.0 | 88.9 (33−100) | 44 | 83.8 ± 15.1 | 88.9 (44−100) | 0.9314 |
|  | After | Course 4 | 49 | 85.3 ± 15.8 | 88.9 (33−100) | 44 | 82.8 ± 16.5 | 83.3 (33−100) | 0.4695 |
| Anxiety | Before | Course 1 | 49 | 69.4 ± 19.1 | 66.7 (33−100) | 44 | 73.5 ± 21.1 | 66.7 (33−100) | − |
|  |  | Course 2 | 49 | 71.4 ± 18.0 | 66.7 (0−100) | 44 | 79.5 ± 19.3 | 66.7 (33−100) | 0.0387 |
|  |  | Course 3 | 49 | 74.1 ± 15.6 | 66.7 (33−100) | 44 | 82.6 ± 19.7 | 100.0 (33−100) | 0.0238 |
|  |  | Course 4 | 49 | 74.8 ± 21.0 | 66.7 (0−100) | 44 | 78.8 ± 21.7 | 66.7 (33−100) | 0.3739 |
|  | After | Course 4 | 49 | 76.9 ± 18.3 | 66.7 (33−100) | 44 | 80.3 ± 21.9 | 83.3 (33−100) | 0.4128 |
| Weight | Before | Course 1 | 49 | 84.4 ± 25.6 | 100.0 (0−100) | 44 | 88.6 ± 16.0 | 100.0 (67−100) | − |
|  |  | Course 2 | 49 | 87.8 ± 18.9 | 100.0 (33−100) | 44 | 88.6 ± 16.0 | 100.0 (67−100) | 0.8097 |
|  |  | Course 3 | 49 | 88.4 ± 18.7 | 100.0 (33−100) | 44 | 88.6 ± 17.5 | 100.0 (33−100) | 0.9576 |
|  |  | Course 4 | 49 | 86.4 ± 19.2 | 100.0 (33−100) | 44 | 88.6 ± 17.5 | 100.0 (33−100) | 0.5589 |
|  | After | Course 4 | 49 | 86.4 ± 17.9 | 100.0 (33−100) | 44 | 87.9 ± 17.7 | 100.0 (33−100) | 0.6894 |
| Sexual interest (men) | Before | Course 1 | 25 | 77.3 ± 20.9 | 66.7 (33−100) | 23 | 79.7 ± 16.6 | 66.7 (67−100) | − |
|  |  | Course 2 | 29 | 79.3 ± 20.7 | 66.7 (33−100) | 23 | 79.7 ± 16.6 | 66.7 (67−100) | 0.9403 |
|  |  | Course 3 | 30 | 78.9 ± 18.5 | 66.7 (33−100) | 23 | 84.1 ± 17.0 | 100.0 (67−100) | 0.3023 |
|  |  | Course 4 | 30 | 81.1 ± 16.8 | 66.7 (67−100) | 23 | 84.1 ± 17.0 | 100.0 (67−100) | 0.5320 |
|  | After | Course 4 | 30 | 81.1 ± 18.9 | 66.7 (33−100) | 23 | 79.7 ± 19.4 | 66.7 (33−100) | 0.7929 |
| Sexual interest (women) | Before | Course 1 | 15 | 100.0 ± 0.0 | 100.0 (100−100) | 17 | 98.0 ± 8.1 | 100.0 (67−100) | − |
|  |  | Course 2 | 17 | 100.0 ± 0.0 | 100.0 (100−100) | 18 | 98.1 ± 7.9 | 100.0 (67−100) | 0.3386 |
|  |  | Course 3 | 17 | 98.0 ± 8.1 | 100.0 (67−100) | 18 | 100.0 ± 0.0 | 100.0 (100−100) | 0.3105 |
|  |  | Course 4 | 17 | 100.0 ± 0.0 | 100.0 (100−100) | 18 | 98.1 ± 7.9 | 100.0 (67−100) | 0.3386 |
|  | After | Course 4 | 17 | 100.0 ± 0.0 | 100.0 (100−100) | 18 | 98.1 ± 7.9 | 100.0 (67−100) | 0.3386 |
| Urinary frequency | Before | Course 1 | 48 | 22.9 ± 21.9 | 16.7 (0−100) | 44 | 23.1 ± 22.5 | 16.7 (0−67) | − |
|  |  | Course 2 | 49 | 21.1 ± 22.8 | 16.7 (0−100) | 44 | 23.5 ± 19.1 | 25.0 (0−67) | 0.5863 |
|  |  | Course 3 | 49 | 20.7 ± 19.7 | 16.7 (0−67) | 44 | 18.6 ± 18.8 | 16.7 (0−67) | 0.5857 |
|  |  | Course 4 | 49 | 20.4 ± 20.2 | 16.7 (0−67) | 44 | 16.3 ± 17.8 | 16.7 (0−67) | 0.3017 |
|  | After | Course 4 | 49 | 19.4 ± 17.8 | 16.7 (0−67) | 44 | 19.3 ± 18.0 | 16.7 (0−67) | 0.9851 |
| Blood and mucus in stool | Before | Course 1 | 49 | 5.1 ± 9.7 | 0.0 (0−33) | 44 | 3.0 ± 7.4 | 0.0 (0−33) | − |
|  |  | Course 2 | 49 | 3.1 ± 8.1 | 0.0 (0−33) | 44 | 2.7 ± 8.0 | 0.0 (0−33) | 0.8070 |
|  |  | Course 3 | 49 | 2.7 ± 7.1 | 0.0 (0−33) | 44 | 2.3 ± 5.8 | 0.0 (0−17) | 0.7409 |
|  |  | Course 4 | 49 | 3.1 ± 8.8 | 0.0 (0−50) | 44 | 2.3 ± 5.8 | 0.0 (0−17) | 0.6149 |
|  | After | Course 4 | 49 | 2.4 ± 6.8 | 0.0 (0−33) | 44 | 1.5 ± 4.9 | 0.0 (0−17) | 0.4860 |
| Stool frequency | Before | Course 1 | 38 | 17.1 ± 24.7 | 8.3 (0−100) | 40 | 20.0 ± 20.7 | 16.7 (0−67) | − |
|  |  | Course 2 | 42 | 16.3 ± 20.0 | 16.7 (0−67) | 41 | 20.3 ± 20.9 | 16.7 (0−67) | 0.3692 |
|  |  | Course 3 | 43 | 14.7 ± 17.1 | 16.7 (0−67) | 41 | 18.3 ± 19.3 | 16.7 (0−67) | 0.3729 |
|  |  | Course 4 | 43 | 13.2 ± 15.2 | 16.7 (0−67) | 41 | 17.1 ± 16.9 | 16.7 (0−67) | 0.2696 |
|  | After | Course 4 | 44 | 13.3 ± 15.9 | 16.7 (0−67) | 41 | 14.2 ± 16.5 | 16.7 (0−67) | 0.7832 |
| Urinary incontinence | Before | Course 1 | 49 | 4.1 ± 11.0 | 0.0 (0−33) | 43 | 7.0 ± 15.5 | 0.0 (0−67) | − |
|  |  | Course 2 | 49 | 6.1 ± 13.0 | 0.0 (0−33) | 44 | 3.0 ± 9.7 | 0.0 (0−33) | 0.2018 |
|  |  | Course 3 | 49 | 7.5 ± 14.1 | 0.0 (0−33) | 44 | 3.8 ± 12.9 | 0.0 (0−67) | 0.1914 |
|  |  | Course 4 | 49 | 6.8 ± 13.6 | 0.0 (0−33) | 44 | 6.1 ± 14.9 | 0.0 (0−67) | 0.8018 |
|  | After | Course 4 | 49 | 6.8 ± 13.6 | 0.0 (0−33) | 44 | 8.3 ± 16.3 | 0.0 (0−67) | 0.6223 |
| Dysuria | Before | Course 1 | 49 | 2.0 ± 8.1 | 0.0 (0−33) | 44 | 6.8 ± 17.0 | 0.0 (0−67) | − |
|  |  | Course 2 | 49 | 2.0 ± 8.1 | 0.0 (0−33) | 44 | 3.0 ± 9.7 | 0.0 (0−33) | 0.5928 |
|  |  | Course 3 | 49 | 1.4 ± 6.7 | 0.0 (0−33) | 44 | 5.3 ± 12.3 | 0.0 (0−33) | 0.0549 |
|  |  | Course 4 | 49 | 0.7 ± 4.8 | 0.0 (0−33) | 44 | 6.1 ± 13.0 | 0.0 (0−33) | 0.0082 |
|  | After | Course 4 | 49 | 0.7 ± 4.8 | 0.0 (0−33) | 44 | 1.5 ± 7.0 | 0.0 (0−33) | 0.5002 |
| Abdominal pain | Before | Course 1 | 49 | 12.9 ± 19.0 | 0.0 (0−67) | 43 | 12.4 ± 17.9 | 0.0 (0−67) | − |
|  |  | Course 2 | 49 | 9.5 ± 15.2 | 0.0 (0−33) | 44 | 12.1 ± 21.7 | 0.0 (0−100) | 0.5019 |
|  |  | Course 3 | 49 | 4.8 ± 11.8 | 0.0 (0−33) | 44 | 15.2 ± 24.3 | 0.0 (0−100) | 0.0092 |
|  |  | Course 4 | 49 | 8.8 ± 16.4 | 0.0 (0−67) | 44 | 11.4 ± 23.8 | 0.0 (0−100) | 0.5497 |
|  | After | Course 4 | 49 | 7.5 ± 14.1 | 0.0 (0−33) | 44 | 12.9 ± 24.1 | 0.0 (0−100) | 0.1848 |
| Buttock pain | Before | Course 1 | 48 | 9.0 ± 16.5 | 0.0 (0−67) | 43 | 13.2 ± 19.4 | 0.0 (0−67) | − |
|  |  | Course 2 | 49 | 8.2 ± 14.5 | 0.0 (0−33) | 44 | 14.4 ± 19.6 | 0.0 (0−67) | 0.0822 |
|  |  | Course 3 | 49 | 8.2 ± 16.0 | 0.0 (0−67) | 44 | 13.6 ± 19.5 | 0.0 (0−67) | 0.1403 |
|  |  | Course 4 | 49 | 8.2 ± 16.0 | 0.0 (0−67) | 44 | 11.4 ± 17.5 | 0.0 (0−67) | 0.3597 |
|  | After | Course 4 | 49 | 6.1 ± 13.0 | 0.0 (0−33) | 44 | 12.9 ± 17.9 | 0.0 (0−67) | 0.0391 |
| Bloating | Before | Course 1 | 49 | 19.0 ± 20.4 | 33.3 (0−67) | 43 | 16.3 ± 22.3 | 0.0 (0−100) | − |
|  |  | Course 2 | 49 | 12.9 ± 17.8 | 0.0 (0−67) | 44 | 15.9 ± 22.1 | 0.0 (0−100) | 0.4734 |
|  |  | Course 3 | 49 | 10.9 ± 17.2 | 0.0 (0−67) | 44 | 15.9 ± 19.7 | 0.0 (0−67) | 0.1921 |
|  |  | Course 4 | 49 | 9.5 ± 16.7 | 0.0 (0−67) | 44 | 11.4 ± 18.9 | 0.0 (0−67) | 0.6195 |
|  | After | Course 4 | 49 | 10.9 ± 17.2 | 0.0 (0−67) | 44 | 10.6 ± 17.3 | 0.0 (0−67) | 0.9382 |
| Dry mouth | Before | Course 1 | 49 | 15.0 ± 18.1 | 0.0 (0−67) | 44 | 6.8 ± 13.6 | 0.0 (0−33) | − |
|  |  | Course 2 | 49 | 18.4 ± 21.6 | 0.0 (0−67) | 44 | 14.4 ± 16.7 | 0.0 (0−33) | 0.3274 |
|  |  | Course 3 | 49 | 17.7 ± 18.1 | 33.3 (0−67) | 44 | 14.4 ± 18.2 | 0.0 (0−67) | 0.3848 |
|  |  | Course 4 | 49 | 12.9 ± 16.4 | 0.0 (0−33) | 44 | 15.2 ± 18.3 | 0.0 (0−67) | 0.5373 |
|  | After | Course 4 | 49 | 12.9 ± 16.4 | 0.0 (0−33) | 44 | 14.4 ± 16.7 | 0.0 (0−33) | 0.6701 |
| Hair loss | Before | Course 1 | 48 | 1.4 ± 6.7 | 0.0 (0−33) | 43 | 0.0 ± 0.0 | 0.0 (0−0) | − |
|  |  | Course 2 | 48 | 3.5 ± 10.3 | 0.0 (0−33) | 44 | 3.8 ± 10.7 | 0.0 (0−33) | 0.8857 |
|  |  | Course 3 | 48 | 2.1 ± 8.2 | 0.0 (0−33) | 44 | 3.8 ± 12.9 | 0.0 (0−67) | 0.4466 |
|  |  | Course 4 | 48 | 4.9 ± 11.9 | 0.0 (0−33) | 44 | 3.8 ± 10.7 | 0.0 (0−33) | 0.6512 |
|  | After | Course 4 | 48 | 4.9 ± 11.9 | 0.0 (0−33) | 44 | 5.3 ± 14.3 | 0.0 (0−67) | 0.8718 |
| Taste | Before | Course 1 | 49 | 3.4 ± 10.2 | 0.0 (0−33) | 44 | 3.0 ± 12.1 | 0.0 (0−67) | − |
|  |  | Course 2 | 49 | 7.5 ± 15.6 | 0.0 (0−67) | 44 | 5.3 ± 12.3 | 0.0 (0−33) | 0.4604 |
|  |  | Course 3 | 49 | 7.5 ± 15.6 | 0.0 (0−67) | 44 | 10.6 ± 20.0 | 0.0 (0−67) | 0.4016 |
|  |  | Course 4 | 49 | 11.6 ± 21.0 | 0.0 (0−67) | 44 | 11.4 ± 22.7 | 0.0 (0−100) | 0.9647 |
|  | After | Course 4 | 49 | 17.0 ± 22.7 | 0.0 (0−67) | 44 | 15.9 ± 24.4 | 0.0 (0−100) | 0.8225 |
| Flatulence | Before | Course 1 | 38 | 14.9 ± 16.8 | 0.0 (0−33) | 40 | 26.7 ± 26.4 | 33.3 (0−100) | − |
|  |  | Course 2 | 42 | 14.3 ± 18.3 | 0.0 (0−67) | 40 | 25.0 ± 19.6 | 33.3 (0−67) | 0.0123 |
|  |  | Course 3 | 43 | 14.7 ± 16.8 | 0.0 (0−33) | 40 | 24.2 ± 21.3 | 33.3 (0−67) | 0.0272 |
|  |  | Course 4 | 43 | 15.5 ± 18.3 | 0.0 (0−67) | 41 | 25.2 ± 22.1 | 33.3 (0−67) | 0.0310 |
|  | After | Course 4 | 44 | 15.9 ± 16.8 | 0.0 (0−33) | 41 | 21.1 ± 17.9 | 33.3 (0−67) | 0.1687 |
| Fecal incontinence | Before | Course 1 | 38 | 6.1 ± 15.2 | 0.0 (0−67) | 40 | 9.2 ± 22.6 | 0.0 (0−100) | − |
|  |  | Course 2 | 42 | 6.3 ± 13.3 | 0.0 (0−33) | 41 | 8.1 ± 19.4 | 0.0 (0−100) | 0.6260 |
|  |  | Course 3 | 43 | 7.0 ± 13.7 | 0.0 (0−33) | 41 | 9.8 ± 20.1 | 0.0 (0−100) | 0.4589 |
|  |  | Course 4 | 43 | 7.8 ± 14.3 | 0.0 (0−33) | 41 | 6.5 ± 13.4 | 0.0 (0−33) | 0.6804 |
|  | After | Course 4 | 44 | 7.6 ± 14.1 | 0.0 (0−33) | 41 | 4.9 ± 11.9 | 0.0 (0−33) | 0.3461 |
| Sore skin | Before | Course 1 | 38 | 11.4 ± 20.9 | 0.0 (0−67) | 40 | 17.5 ± 25.0 | 0.0 (0−100) | − |
|  |  | Course 2 | 42 | 9.5 ± 16.9 | 0.0 (0−67) | 41 | 19.5 ± 23.5 | 0.0 (0−67) | 0.0290 |
|  |  | Course 3 | 43 | 9.3 ± 16.8 | 0.0 (0−67) | 41 | 16.3 ± 22.5 | 0.0 (0−67) | 0.1110 |
|  |  | Course 4 | 43 | 7.8 ± 16.0 | 0.0 (0−67) | 41 | 13.8 ± 18.2 | 0.0 (0−67) | 0.1082 |
|  | After | Course 4 | 44 | 7.6 ± 15.9 | 0.0 (0−67) | 41 | 13.0 ± 16.5 | 0.0 (0−33) | 0.1250 |
| Embarrassment | Before | Course 1 | 38 | 1.8 ± 7.5 | 0.0 (0−33) | 40 | 3.3 ± 10.1 | 0.0 (0−33) | − |
|  |  | Course 2 | 42 | 1.6 ± 7.2 | 0.0 (0−33) | 41 | 3.3 ± 10.0 | 0.0 (0−33) | 0.3859 |
|  |  | Course 3 | 43 | 3.1 ± 9.8 | 0.0 (0−33) | 41 | 4.9 ± 11.9 | 0.0 (0−33) | 0.4567 |
|  |  | Course 4 | 43 | 2.3 ± 8.6 | 0.0 (0−33) | 41 | 4.9 ± 11.9 | 0.0 (0−33) | 0.2621 |
|  | After | Course 4 | 44 | 3.8 ± 10.7 | 0.0 (0−33) | 41 | 4.9 ± 11.9 | 0.0 (0−33) | 0.6581 |
| Stoma care problems | Before | Course 1 | 2 | 16.7 ± 23.6 | 16.7 (0−33) | 2 | 33.3 ± 0.0 | 33.3 (33−33) | − |
|  |  | Course 2 | 2 | 16.7 ± 23.6 | 16.7 (0−33) | 2 | 16.7 ± 23.6 | 16.7 (0−33) | 1.0000 |
|  |  | Course 3 | 2 | 16.7 ± 23.6 | 16.7 (0−33) | 2 | 33.3 ± 0.0 | 33.3 (33−33) | 0.4226 |
|  |  | Course 4 | 2 | 16.7 ± 23.6 | 16.7 (0−33) | 2 | 16.7 ± 23.6 | 16.7 (0−33) | 1.0000 |
|  | After | Course 4 | 2 | 16.7 ± 23.6 | 16.7 (0−33) | 2 | 33.3 ± 0.0 | 33.3 (33−33) | 0.4226 |
| Impotence | Before | Course 1 | 25 | 14.7 ± 21.7 | 0.0 (0−67) | 22 | 27.3 ± 33.6 | 16.7 (0−100) | − |
|  |  | Course 2 | 28 | 19.0 ± 23.0 | 0.0 (0−67) | 23 | 23.2 ± 27.4 | 33.3 (0−100) | 0.5600 |
|  |  | Course 3 | 29 | 18.4 ± 22.9 | 0.0 (0−67) | 23 | 23.2 ± 29.2 | 0.0 (0−100) | 0.5091 |
|  |  | Course 4 | 29 | 17.2 ± 24.6 | 0.0 (0−100) | 23 | 17.4 ± 24.4 | 0.0 (0−67) | 0.9826 |
|  | After | Course 4 | 29 | 12.6 ± 18.7 | 0.0 (0−67) | 23 | 23.2 ± 34.0 | 0.0 (0−100) | 0.1610 |
| Dyspareunia | Before | Course 1 | 12 | 0.0 ± 0.0 | 0.0 (0−0) | 11 | 6.1 ± 13.5 | 0.0 (0−33) | − |
|  |  | Course 2 | 15 | 0.0 ± 0.0 | 0.0 (0−0) | 13 | 5.1 ± 12.5 | 0.0 (0−33) | 0.1236 |
|  |  | Course 3 | 17 | 3.9 ± 11.1 | 0.0 (0−33) | 14 | 4.8 ± 12.1 | 0.0 (0−33) | 0.8416 |
|  |  | Course 4 | 19 | 1.8 ± 7.7 | 0.0 (0−33) | 14 | 0.0 ± 0.0 | 0.0 (0−0) | 0.3992 |
|  | After | Course 4 | 21 | 4.8 ± 12.0 | 0.0 (0−33) | 14 | 0.0 ± 0.0 | 0.0 (0−0) | 0.1475 |

Abbreviations: EORTC QLQ-C30 and QLQ-CR29, European Organisation for Research and Treatment of Cancer Quality of Life Questionnaire module for all cancer patients (QLQ-C30) and for colorectal cancer patients (QLQ-CR29); SD, standard deviation; QoL, Quality of Life

**Supplemental Table 5. Completion rates of 4 courses of capecitabine treatment without delay or dose reduction**

|  | Cystine/theanine  (n = 49) | Placebo  (n = 45) | Difference between groups  Point estimation [90% CI], *p*-value |
| --- | --- | --- | --- |
| Completion rate  No. of patients (%) | 43 (87.8) | 38 (84.4) | 3.31 [-10.70, 17.32], 0.6423 |
| Completion rate without delay or reduction  No. of patients (%) | 34 (69.4) | 30 (66.7) | 2.72 [-16.15, 21.60], 0.7774 |

**Supplemental Table 6. Time to completion of 4 courses of capecitabine treatment**

|  | Cystine/theanine (n = 43) | Placebo (n = 38) | Difference between groups  Point estimation [90% CI], *p*-value |
| --- | --- | --- | --- |
| Time to completion (days) | 85.0 | 83.4 | 1.60 [-1.98, 5.18], 0.4583 |

**Supplemental Table 7. Total dose of capecitabine treatment**

|  | Cystine/theanine (n = 43) | Placebo (n = 38) | Difference between groups  Point estimation [90% CI], *p*-value |
| --- | --- | --- | --- |
| Total dose (×10^4^ mg) | 22.0 | 20.8 | 1.21 [-0.71, 3.12], 0.2975 |
